# A synthetic cyclic peptide for promoting antigen presentation and immune activation

**DOI:** 10.1038/s41541-024-01050-4

**Published:** 2025-01-15

**Authors:** Jiahui Zhang, Harrison Y. R. Madge, Asmaa Mahmoud, Lantian Lu, Wanyi Wang, Wenbin Huang, Prashamsa Koirala, Jazmina L. Gonzalez Cruz, Wei Yang Kong, Sahra Bashiri, Ahmed O. Shalash, Waleed M. Hussein, Zeinab G. Khalil, James W. Wells, Istvan Toth, Rachel J. Stephenson

**Affiliations:** 1 School of Chemistry and Molecular Biosciences, The University of Queensland, Brisbane, Australia; 2 Faculty of Medicine, Frazer Institute, The University of Queensland, Brisbane, Australia; 3 Institute for Molecular Bioscience, The University of Queensland, Brisbane, Australia; 4 School of Pharmacy, The University of Queensland, Brisbane, Australia

**Keywords:** Adjuvants, Peptide vaccines

## Abstract

Cyclic peptides are often used as scaffolds for the multivalent presentation of drug molecules due to their structural stability and constrained conformation. We identified a cyclic deca-peptide incorporating lipoamino acids for delivering T helper and B cell epitopes against group A *Streptococcus* (GAS), eliciting robust humoral immune responses. In this study, we assessed the function-immunogenicity relationship of the multi-component vaccine candidate (referred to as VC-13) to elucidate a mechanism of action. We identified a potential universal delivery platform, not only capable of adjuvanting different peptide epitopes (e.g., NS1 and 88/30 from group A *Streptococcus*, gonadotropin hormone releasing hormone [GnRH]), but also protein antigens (e.g., bovine serum albumin [BSA], receptor binding domain (RBD) of the SARS-CoV-2 protein responsible for COVID-19 infection [SARS-CoV-2 RBD]) and small molecular haptens (e.g., cocaine). All vaccine candidates self-assembled into sub-500 nm nanoparticles and induced high antigen-specific systemic IgG titers and opsonic potential compared to the antigen co-administered with a commercial adjuvant, complete Freund’s adjuvant. Notably, presence of the cyclic decapeptide in this vaccine increased accumulation in the draining inguinal lymph nodes, facilitating cellular uptake of peptide antigens. Furthermore, the lipoamino acid promoted dendritic cell activation, acting as both toll-like receptors 2 and 4 -targeting moiety. Our study revealed the importance of the cyclic decapeptide and lipoamino acid presence in antigen presentation and immune response activation, leading onto the development of a fully synthetic, self-assembled, and promising platform for the delivery of subunit vaccines and anti-drug vaccines.

## Introduction

Adjuvants, also known as immunopotentiators, are essential components of vaccines that stimulate an optimum immune response towards a specific antigen, creating an immunological memory^1^. The development of vaccines and other immunotherapies has been challenged by the use of heterogeneous immune adjuvants whose mechanism is not well understood, leading to untargeted antigen display. Synthetic peptides make excellent antigens due to their known chemical characterisation leading to the generation of a specific immune response. However, peptide antigens are poorly immunogenic and require co-administration with strong adjuvants to be effective as a vaccine. Many adjuvants have been investigated for peptide vaccines, including particulates, oil emulsions, toll-like receptor ligands, immunostimulating complexes, and other biologically-derived materials (e.g., squalene, chitosan, alginates, and saponins)^2,3^. These adjuvants comprise chemically or structurally heterogenous materials, making their characterisation, mechanism of understanding, and regulatory approval somewhat more challenging, and it is these factors that are driving the development of adjuvant-free delivery systems for subunit vaccines.

Before 1997, insoluble aluminum hydroxide, more commonly known as alum, was the only licensed adjuvant for pediatric subunit vaccines. In 1997, the first oil-in-water emulsion adjuvant, MF59, was approved in Europe for use in vaccines for adults. At present, there are only a handful of licensed adjuvants available for use in human vaccines (e.g., alum, MF59, AS0, CPG-1018)^4,5^. Vaccines that contain alum are known to generate a depot effect following vaccine administration, mimicking the sustained expression of pathogen associated molecular patterns in traditional attenuated vaccines. However, the rapid injection of alum leads to tissue damage and other unwanted side effects^6–8^.

Self-assembled peptides have been explored for a variety of applications, including biomedical and biotechnological applications, providing several advantages ranging from synthetic definition, molecular specificity, and control over the nanoscale positioning of ligands. In our laboratory, we have investigated cyclic lipo-/peptides as antigen vaccine scaffolds and adjuvant systems for the delivery of group A *Streptococcus* (GAS) peptide antigens^9–11^. With the improved display of antigens, lipo-/cyclic peptides have been shown to enhance the pharmacokinetic properties (e.g., reduced proteolytic degradation) of antigens and have demonstrated self-adjuvanting ability (e.g., carbohydrate antigens in cancer models)^12^.

Through extensive structure-activity studies, we have demonstrated that a physical mixture of a cyclic decapeptide, toll-like receptor 2 (TLR-2) targeting lipid peptide and GAS B cell epitope (J8) chemically-conjugated to a universal T helper cell epitope (PADRE) produced significant IgG antibodies when administered subcutaneously to mice with strong opsonisation potential against clinical GAS^9–11^. We then went on to show the orientation of the vaccine B and T helper epitopes played an important role in the immune response obtained, where an exposed C-terminus of the GAS B cell epitope provided the optimum results and assessed different lipids as part of the adjuvant formulation to conclusively show that a 16-carbon alpha-amino fatty acid lipid was required for strong immunoreactivity. Interestingly, physicochemical assessment of the vaccines proved the alpha helix structure of the GAS B cell epitope was retained, impacting particle self-assembly and vaccine immunoreactivity, where the cyclic decapeptide and lipid were essential for strong immunological responses in a murine vaccine model^9–11^.

**Fig. 1:**
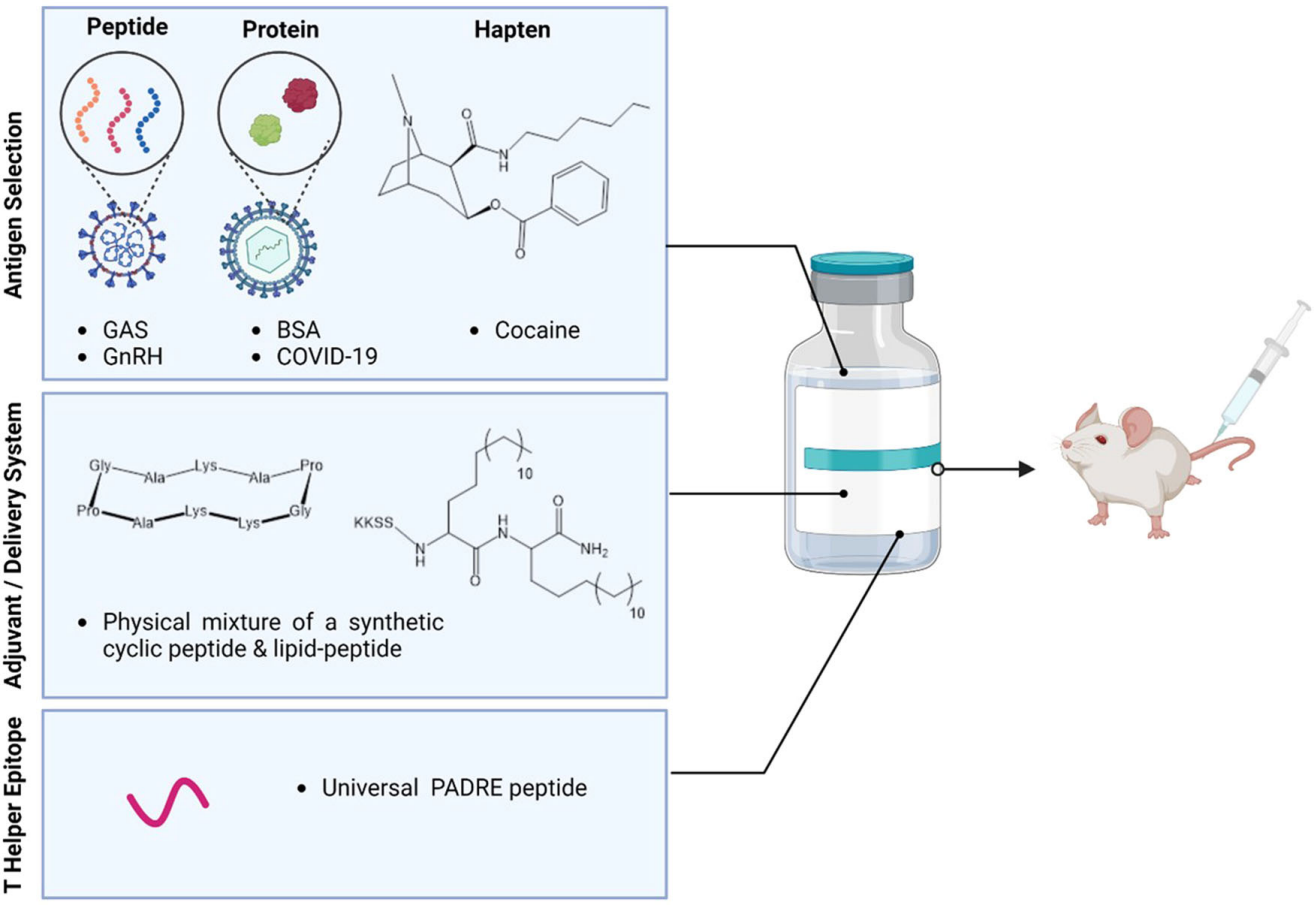
Schematic illustration of the cyclic peptide-based nanovaccine and its components. The nanovaccine consists of an adjuvant/delivery system (lipid plus cyclic decapeptide), peptide antigens (three B cell peptide epitopes from GAS and GnRH), protein antigens (BSA and SARS-CoV-2 RBD), and a cocaine hapten. These components were all assessed in mice as a four-part study. Created in BioRender. Stephenson, R. (2025) https://BioRender.com/x15y964.

In the present work, we sought to determine if a synthetic adjuvant formulation comprised of a cyclic decapeptide physically mixed with a short lipopeptide enhanced the immunology of three different types of antigens, including peptide, protein, and a synthetic hapten (Fig. 1).

The peptide antigens (NS1, 88/30 and J8) assessed in this study were derived from the GAS M protein. GAS is the principal etiologic agent of both rheumatic fever and rheumatic heart disease, the two major postinfectious complications responsible for a large proportion of the over 320,000 GAS-related deaths reported annually^13^. This statistic makes GAS one of the major human pathogens, only being exceeded by *Plasmodium falciparum*, *Streptococcus pneumoniae*, *Mycobacterium tuberculosis*, and human immunodeficiency virus (HIV)^14^. GAS also represents a major health burden in Australia, with rates of persistent infection as high as 45% of the population in some remote Indigenous communities. Subunit vaccine design against GAS is driven by the complications faced with whole protein vaccination where proteins from GAS attack the human heart, causing rheumatic heart disease^15^. Specifically, in this study through in vivo and in vitro assessments, we evaluated the ratio of antigen (B cell peptide derived from the GAS antigen) to adjuvant (cyclic peptide plus lipopeptide) ratio with the aim to reduce the required number of vaccinations necessary for protection. Leading on from this, the adjuvant potential against two different GAS peptide antigens, as well as a gonadotropin hormone releasing hormone (GnRH) peptide antigen (Fig. 1) were also investigated.

GnRH has been intensively studied as a target for the control of fertility and hormone dependent cancers. In most studies, a decapeptide (EHWSYGLRPG), which is identical to the native GnRH sequence has been used. Here, we assessed the translational adjuvant capacity towards this GnRH decapeptide antigen (Fig. 1).

It is well known that protein-based antigens need assistance from adjuvants to drive a stronger, targeted immune response^16,17^. A recent example of this is the Novavax vaccine, comprised of the coronavirus spike protein co-administered with the Matrix-M adjuvant. We further assessed in vivo the translational adjuvant ability of our adjuvant with protein antigens, including bovine serum albumin (BSA) and the receptor binding domain (RBD) of the SARS-CoV-2 protein responsible for COVID-19 infection (SARS-CoV-2 RBD) (Fig. 1).

With no pharmacological treatment for cocaine addiction, anti-cocaine vaccines are seen as an attractive way to bind free cocaine before it passes through the blood-brain barrier and into the brain, providing a ‘rewarding’ effect for the cocaine user^18^. This ‘reward’ drives cocaine use, and quenching this ‘reward’ is thought to assist addicts to overcome their addiction^18^. Lastly, we investigated the adjuvant ability of a small synthetic hapten, cocaine, using both in vitro and in vivo assessment (Fig. 1).

These studies indicated that a surprisingly robust adjuvant response was generated against all three systems (peptide, protein, and small hapten), demonstrating that this cyclic decapeptide co-mixed with lipo-peptide serves as a powerful, chemically defined adjuvant. Notably, increasing the adjuvant ratio of the cyclic decapeptide co-mixed with lipo-peptide was shown to lead to strong antibody titres and opsonic protection against GAS clinical isolates after a single immunisation.

## Results

### Optimization of Adjuvant-to-Antigen Ratios Enhances Humoral Immune Responses

The optimisation of the adjuvant ratio of the cyclic decapeptide and lipid to antigen (peptide) ratio (up to 8× adjuvant to antigen ratio compared with the initial assessment) as a physically mixed vaccine was assessed (Table 1)^11^.

**Table 1:** Schematic illustration of vaccines assessed for an increase in adjuvant to antigen ratio

Vaccine Name	Adjuvant	Antigen
VC-13		
VC-16	Adjuvant ratio increased by 2×	
VC-17	Adjuvant ratio increased by 4×	
VC-18	Adjuvant ratio increased by 8×	
PADRE-J8 + CFA	CFA	

**J8** B cell antigen (QAEDKVKQSREAKKQVEKALKQLEDKVQ); **PADRE** T helper epitope (AKFVAAWTLKAAA). Positive (**PADRE-J8** + **CFA**) and negative (**PBS**) controls were used. ‘**VC**’ represents ‘vaccine candidate’.

Female C57BL/6 mice were immunised subcutaneously at the tail base on days 0, 21, 28, and 35 with 30 μg of **VC-13** (or an increased adjuvant-normalised dose according to Table 1). The negative control was PBS, and the positive control was PADRE-J8 **[BB3]** (30 μg) emulsified in 50:50 PBS: CFA primary immunisation, with **BB3** (30 μg) in PBS for boosts. Blood was collected from the tail tip 7 days following each injection before serum was analysed for total antigen-specific serum IgG antibodies by ELISA.

**Fig. 2:**
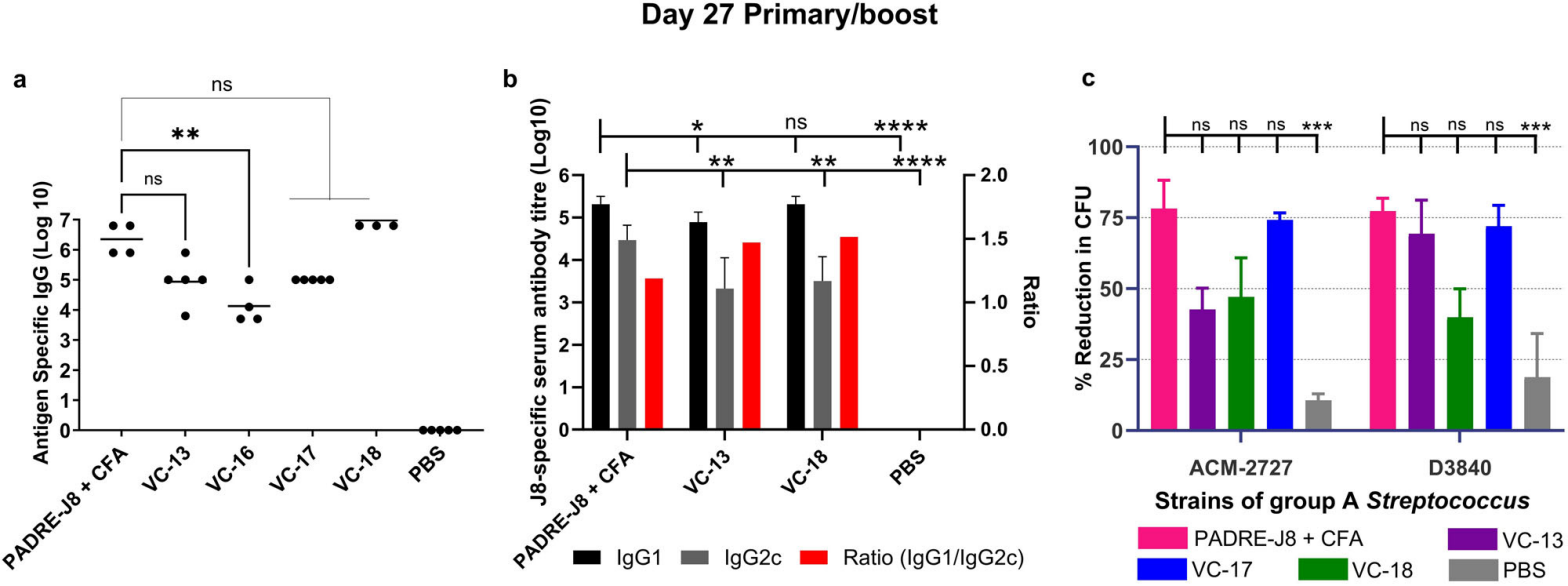
J8-specific humoral immune responses at day 27 following subcutaneous immunisation in C57BL/6 mice. Mice (*n* = 5 per group) were immunised with the vaccine candidates (Table 1), the negative control (PBS) and the positive control (PADRE-J8 + CFA). **a** J8-specific total IgG titres (log 10) at day 27 as determined by ELISA. Each point represents an individual mouse and bars represent the average antigen-specific serum IgG antibody titres. **b** J8-specific average IgG1 and IgG2c titres (log 10) and IgG1/IgG2c ratio at day 27 as determined by ELISA. **c** Average opsonisation percentage of different GAS strains by serum collected on day 27. Results are represented as opsonisation percentage compared to untreated reference wells, with error represented as the standard error of the mean (SEM). Statistical analysis was performed using a one-way ANOVA followed by Tukey post-hoc test (ns, *p* > 0.05; *, *p* < 0.05; **, *p* < 0.01; ***, *p* < 0.001; ****, *p* < 0.0001).

On day 27 (primary immunisation plus one boost), we observed significantly high IgG titres of **VC-17** (four times the adjuvant ratio of **VC-13**; Table 1) and **VC-18** (eight times the adjuvant ratio of **VC-13**; Table 1), which was able to produce a high level of J8-specific IgG antibodies with no significant difference to the positive control (**PADRE-J8** + **CFA**) (Fig. 2a). Interestingly, **VC-13** (our original vaccine formulation)^9–11^ also showed no significant difference in antibody titre to that of the positive control on day 27. Notably, on day 34 (primary immunisation plus two boosts), we observed a significantly high potency of all vaccines at all ratios tested (Fig. S13a). No significant difference was observed on day 41 for all vaccines (Fig. S13b). C57BL/6 mice produce four distinct IgG subclasses: IgG1, IgG2b, IgG2c, and IgG3, with IgG2c serving as a critical indicator of Th1-type immune responses and IgG1 primarily associated with Th2-type responses^19^. The ratio of IgG2c/IgG1 provides insights into the balance between Th1 and Th2 response, eliciting longer-lasting immunity and enhancing protection through both humoral and cell-mediated mechanisms. To further validate the differential immune responses elicited by vaccine candidates, we analysed the IgG subclass distribution (IgG1 and IgG2c) for select groups, including PADRE-J8 + CFA, VC-13, VC-18, and PBS. Here, all groups induced the most balanced immune response with a Th2/Th1 ratio of <1.5 (Fig. 2b). Whereas VC-13 and VC-18 showed a stronger Th2-biased immune response with a higher Ig1/IgG2c ratio than positive control.

In a mouse challenge model, the reduction in colony counts of GAS bronchial and cutaneous strains has been associated with high antibody titres^9,20^. Notably, the opsonic activity of these vaccines shows **VC-18** to have the strongest opsonisation ability against two different clinical isolates of GAS (Fig. 2c). Excitingly, this indicated that a reduction in the number of vaccine doses was achieved following an increase in adjuvant ratio (8×) when used as a vaccine against GAS infection with the J8 B cell peptide antigen.

**Fig. 3:**
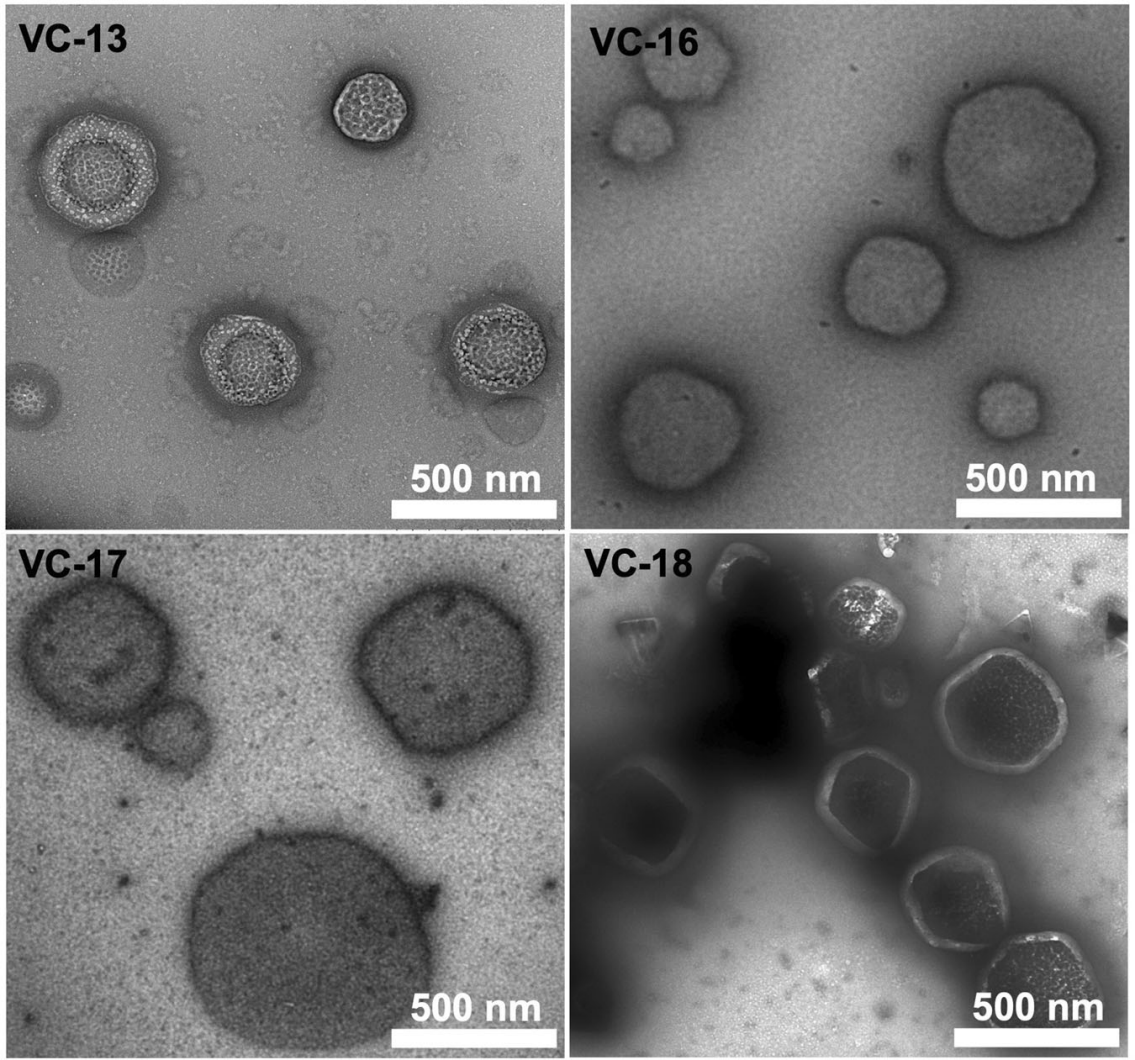
TEM of VC-13, VC-16, VC-17 and VC-18 (0.1 mg/mL). Rulers indicate a scale of 500 nm. Negative staining was performed using 2% uranyl acetate.

The size and morphology of the vaccine candidates were analysed using transmission electron microscopy (TEM) (Fig. 3). Here, large particle aggregates were detected on images of **VC-13**, **VC-16**, **VC-17**, and **VC-18** which were of a typical spherical structure (100–500 nm in diameter) without visible traces of large aggregates.

### Cyclic Lipopeptide Facilitates Lymphatic Targeting and Antigen Uptake

We evaluated the in vivo delivery capability of **VC-13** in C57BL/6J mice. To visualise the organ distribution of our formulations, **BB3**, KKSSC16C16 [**BB4**], and the cyclic decapeptide [**BB6**] were fluorescently labelled with Cyanine5.5 (Cy5.5) using the copper-catalysed click reaction and subjected to fluorescence imaging analysis via subcutaneous injection^21,22^. Following immunisation at the mice tail base, organs (lymph nodes, spleen, kidneys, liver, heart, and lung) were harvested at 4 hr, 8 hr, 12 hr, and 24 hr and reported as the fluorescent percentage according to image the fluorescene intensity (651 nm excitation and 670 nm emission) by PerkinElmer IVIS (Fig. S14).

Robust fluorescently labelled peptide accumulations were readily detected traveling up the muscle tissue from the injection site for all groups except the negative control, PBS. Only trace amounts of labelled compounds were found in the heart, lungs, kidneys, and spleen. At 4 hr post administration, 60% of labelled **BB3** peptide antigen was present in the liver and 20% in the draining lymph node anatomical regions. Over 8 hr, the signal increased in the lymph nodes by an average of 40%, whereas **Cy5.5-VC-13** reached the same amount 24 hr post immunisation, predominantly aggregating in the liver. Notably, the addition of adjuvants contributed to alterations in the delivery of the peptide antigen, leading to an extended residence time in the liver followed by further distribution to lymphoid organs. Furthermore, the high fluorescence in the liver was attributed to the pathway of hepatobiliary clearance, responsible for the removal of foreign pathogens from circulation^23^. Moreover, the Cy5.5-peptide conjugate was more prominently eliminated through hepatic clearance rather than renal clearance due to their chemical properties^24^. Notably, in less than 4 hr post injection, the labelled cyclic peptide predominantly localised in the liver and inguinal lymph nodes and continued to accumulate there for at least 24 hr post-injection. Recent studies have shown that lipid nanoparticle formulations had targetability into the liver post-administration^25^.

**Fig. 4:**
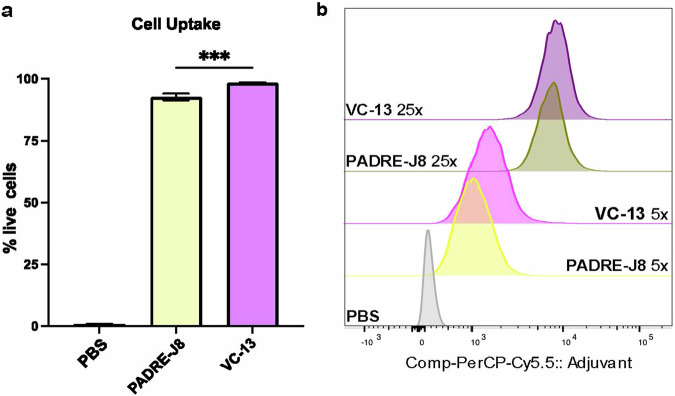
Concentration-dependent uptake of Cy5.5-BB3 in DC2.4 cells. **a** Percentage of fluorescence-positive DC2.4 cells after treatment with Cy5.5-labelled **BB3** or VC-13 at a concentration of 5× (0.5 μM). **b** Mean Fluorescence Intensity (MFI) of DC2.4 cell uptake at two concentrations: 5× (0.5 μM) and 25× (2.5 μM), compared with PBS as a negative control. Statistical significance was determined using a one-way ANOVA (***, *p* < 0.001).

Following 4 hr incubating DC2.4 cells with fluorescently labeled compounds **Cy5.5-BB3**, **Cy5.5-BB4**, **Cy5.5-BB6** and **Cy5.5-VC-13**, cellular uptake of these vaccine antigens was positively correlated with concentration and saturated at 25× (2.5 μM) (Fig. 4a, b). A significant difference in fluorescence signal intensity between the unadjuvanted antigen and **VC-13** at an equivalent 5× (0.5 μM) antigen concentration was also observed (Fig. 4a). The efficient cell uptake represented the enhanced presentation of antigen to the immune system, triggering an immune response and the production of antibodies. Here, it was demonstrated that the chemical properties of the fluorescent dye have an impact on targeting efficiency, especially the lipophilicity and charge presented by Cy5.5 in in vitro uptake experiments^24^.

### Dendritic Cell Maturation and Toll-Like Receptor Activation Induced by VC-13

**Fig. 5:**
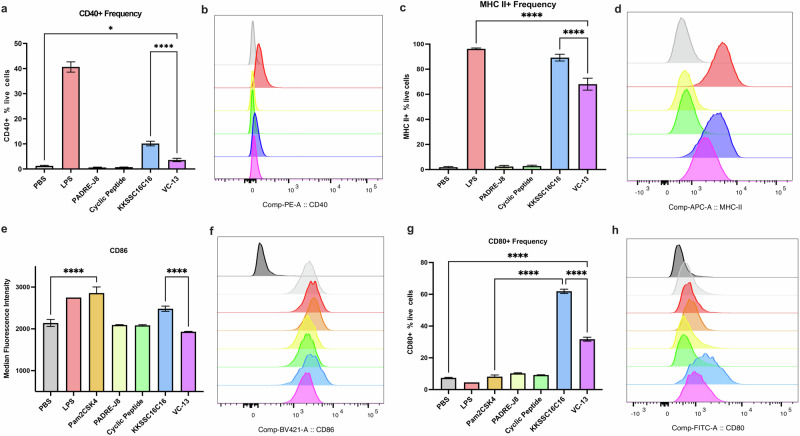
In vitro maturation status of DC2.4 cells after 24 hr treatment with VC-13 and its components. The efficacy of VC-13 was evaluated by analysing the expression of surface markers CD40, MHC II, CD80, and CD86 using flow cytometry. **a**, **b** Percentage and MFI of CD40 expression in live DC2.4 cells. **c**, **d** Percentage and MFI of MHC II expression in live DC2.4 cells. **e**, **f** MFI and histograms of CD86 expression in live DC2.4 cells. **g**, **h** Percentage and MFI of CD80 expression in live DC2.4 cells. The histograms in (**f**) and (**h**) include a fluorescence minus one (FMO) signal represented by dark grey histograms. Histogram colours correspond to the samples in the charts. Statistical significance was determined using a one-way ANOVA (*, *p* < 0.05; ****, *p* < 0.0001).

To assess if the cyclic decapeptide and lipid (adjuvant) enhanced dendritic cell maturation in vitro, PBS (negative control), lipopolysaccharide (LPS) for TLR-4 ligand (positive control), Pam_2_Cys-Ser-(Lys)_4_ (Pam2CSK4) for TLR2/TLR-6 ligand (positive control), cyclic decapeptide [**BB6**], KKSSC16C16 [**BB4**], PADRE-J8 **[BB3]**, and **VC-13** were incubated with DC2.4 cells, followed by flow cytometry analysis after 24 hr of treatment (Fig. 5, Fig. S15). The expression of CD80, CD86, CD40 and MHC II were selected as activation markers to indicate DC maturation^26^. Although CD80 and CD86 are considered crucial markers for assessing cell maturation status, inherent elevated levels of CD86 expression were observed in DC2.4 cells as reported (Fig. 5e, f)^26^. Despite this, the expression of CD40, MHC II, CD80 and CD86 were obviously up regulated upon the addition of **BB4**, especially surpassing that induced by LPS and Pam2CSK4 (Fig. 5). Like PBS, neither **BB3** nor cyclic peptide induced the expression of surface markers. Interestingly, VC-13 trended towards significantly lower expression of all tested markers compared to **BB4**, but still presented significant cell maturation in upregulating the selected markers compared with naked antigen conjugate **BB3**. The differences in surface marker expression patterns reflect varying maturation statuses of cells activated by different samples^26^. Specifically, VC-13 induced and delayed dendritic cell maturation in comparison to **BB4** at the timepoint 24 hr.

**Fig. 6:**
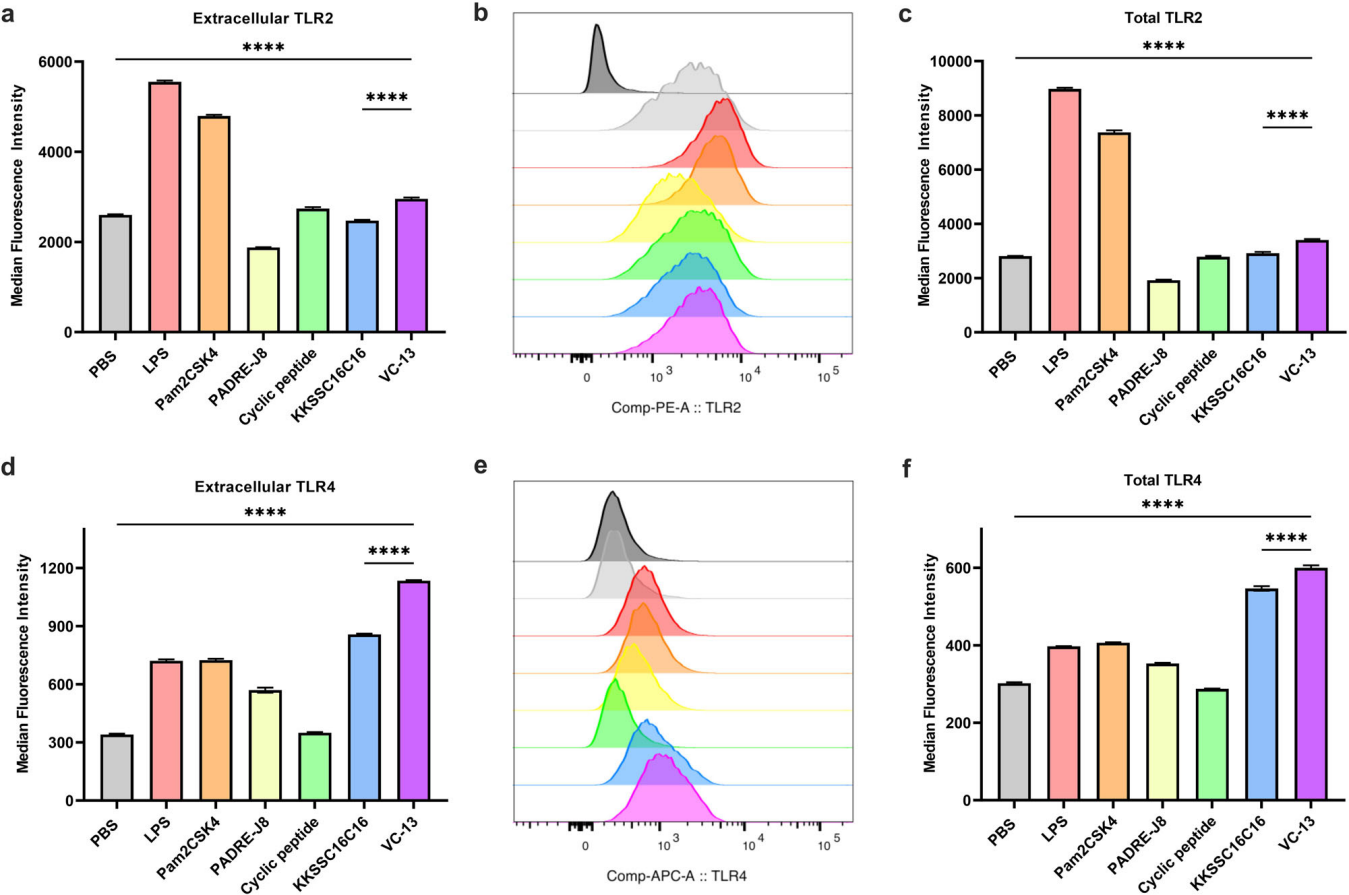
TLR2 and TLR4 activation in DC2.4 cells following 24 hr treatment. TLR2 and TLR4 activation was assessed in vitro using flow cytometry, evaluating extracellular and intracellular expression. **a**, **b** MFI and histograms of TLR2 activation in live DC2.4 cells. **c** MFI of TLR2 expression in DC2.4 cells, showing extracellular and intracellular (total) expression. **d**, **e** MFI and histograms of TLR4 activation in live DC2.4 cells. **f** MFI of TLR4 expression in DC2.4 cells, showing extracellular and intracellular (total) expression. Statistical significance was determined using a one-way ANOVA (****, *p* < 0.0001).

To further determine the mechanism of action of **BB4** and **BB6**, all components of VC-13-induced stimulation via TLR2 and TLR4 was tested in DC2.4 cells extracellularly and intracellularly. Figure 6a, b shows the expression of TLR2 and Fig. 6d, e shows the significant high expression of TLR4 of **BB4** and **VC-13** showed. The significant upregulation of total TLRs in Fig. 6c, f also proved the activity of TLR2 and 4-mediated endocytosis respectively within the cells after 24 hr incubation, **BB4**, was mediated by TLR2 and TLR4 receptors on the surface of dendritic cells and promoted the process of antigen recognition and internalization, ultimately leading to an innate immune response (Fig. 6). This suggested that receptor-mediated endocytosis played an important role in the presentation of **VC-13**. Also, there is no proof that TLR2 and TLR4 have the potential of forming a heterodimer as co-receptor^27^. Liliana et al. showed biglycan as an endogenous ligand has the potential to activate both TLR2 and TLR4 in macrophages, leading to stimulate the pathway of p38, ERK and NF-KB and mediate innate immunity^28^.

### Broad Immunogenicity of the Cyclic Lipopeptide Platform for Alternative Peptide Antigens of Group A *Streptococcus* and GnRH

Peptide antigens are comprised of a chain of amino acids, where the composition of this amino acid chain determines the overall charge, secondary structure, folding, etc. These aspects lead to changes in how the peptide antigen interact with an adjuvant in solution, particle formation, and therefore immune response^29^. Here, assessment of different peptide antigens which have different compositions allow us to observe how effective the adjuvant is. Here, two different GAS antigens (88/30 and NS1) were chosen to achieve this goal and were compared alongside the J8 peptide previously reported^9,10,20^. Assessment of a GnRH peptide, intensively studied as a target for the control of fertility and hormone dependent cancers, was also undertaken. Table 2 outlines the different properties of the peptides assessed, including their charge, antigen length, sequence, and secondary structure. Also included in Table 2 are the properties of the cyclic decapeptide and T helper peptide (PADRE).

**Table 2:** GAS peptide antigen properties

Peptide Antigen	Peptide Sequence	Sequence Length/% Ratio Hydrophilic Residues	Net Charge at pH 7.0	Predicted Secondary Structure*	Isoelectric Point
J8	NH_2-_QAEDKVKQSREAKKQVEKALKQLEDKVQ-CONH_2_	28/71	3	Alpha helix: 82% Coil: 18%	10.19
NS1	NH_2-_RVTTRSQAQDAAGLKEKAD-CONH_2_	19/53	2	Coil: 100%	10.63
88/30	NH_2-_DNGKAIYERARERALQELGP-CONH_2_	20/50	1	Alpha helix: 75% Coil: 25%	9.66
GnRH	NH_2-_EHWSYGLRPG-CONH_2_	10/30	1	Coil: 100%	9.85
PADRE	NH_2-_AKFVAAWTLKAAA-CONH_2_	13/15	3	Coil: 23% Beta sheet: 77%	*Not determined*
Cyclic decapeptide	-AK_(alkyne)_APGKK_(alkyne)_APG-	10/30	1	** Alpha helix: 2.7% Beta sheet: 26.4% Beta turn: 19.4% Coil: 51.5%	*Not determined*
Lipid peptide	NH_2_-**KKSS-C16-C16**-CONH_2_	6/66	3	** Beta sheet: 45.8% Beta turn: 13.5% Coil: 40.7%	*Not determined*

^*^ Predicted using PROTEUS2 software; ** reported previously^9^.

Peptide antigens assessed displayed a range of properties, including different lengths of sequence (range from 10 to 28), net charge (1–3), hydrophilic amino acid ratio (30–71%), and secondary structure (Table 2). These properties all contributed to the effectiveness of the peptides as antigens, and their formulation with the adjuvant to form vaccine nanoparticles.

A structure-activity study was performed assessing the adjuvant activity of NS1 and 88/30 vaccine antigens conjugated to the C- and N-terminus of the T helper PADRE peptide and compared to commercially-adjuvanted (CFA) controls (Table S1). The GnRH antigen was only conjugated to the N-terminus of the T helper epitope (Table S1).

**Fig. 7:**
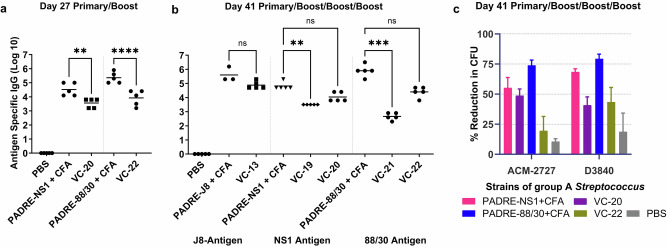
Antigen-specific total IgG titres and opsonization activity induced by subcutaneous immunisation. **a** Total IgG titre (log 10) specific to J8, 88/30, or NS1 on day 27, following primary immunisation and one boost, as determined by ELISA. **b** Total IgG titres (log 10) on day 41, following primary immunisation and three boosts, measured by ELISA. Each point represents an individual mouse, with bars indicating the average antigen-specific serum IgG antibody titres. **c** Average opsonisation percentage of various GAS strains by serum collected on day 41 from immunised mice. Results are represented as the opsonisation percentage relative to reference untreated wells, with error represented as the SEM. Mice (*n* = 5 per group) were immunised with vaccine candidates (Table S1), negative control (**PBS**) and positive controls (**antigen-PADRE** + **CFA**). Statistical analysis was performed using a one-way ANOVA followed by Tukey post-hoc test (ns, *p* > 0.05; **, *p* < 0.01; ***, *p* < 0.001; ****, *p* < 0.0001).

Using the same in vivo experimental conditions reported above, an IgG ELISA on day 41 showed no significant difference between the PADRE C-terminally conjugated antigens (**VC-20** and **VC-22**) and their respective positive controls (Fig. 7). This was consistent with that of the J8 peptide (**VC-13**; Fig. 7), which also had a stronger immune response when conjugated to the C-terminus of the PADRE T helper peptide. Notably, on day 27, antigen-specific IgG titres for **VC-20** (88/30) were stronger than that of **VC-22** (NS1) (Fig. 7), which was also reflected in the visual assessment of day 41 titres for these vaccines, where although non-significant, the IgG titre for the **VC-20** vaccinated mice was higher than that of the **VC-22** mice. However, the opsonisation activity of **VC-22** on day 41 was significantly reduced compared with that of **VC-20** (*p* = 0.0005 in ACM-2727; *p* = 0.0185 in D3840) (Fig. 7). Antigen alone (J8, NS1, 88/30) has previously shown negligible antibody production^30,31^.

**Fig. 8:**
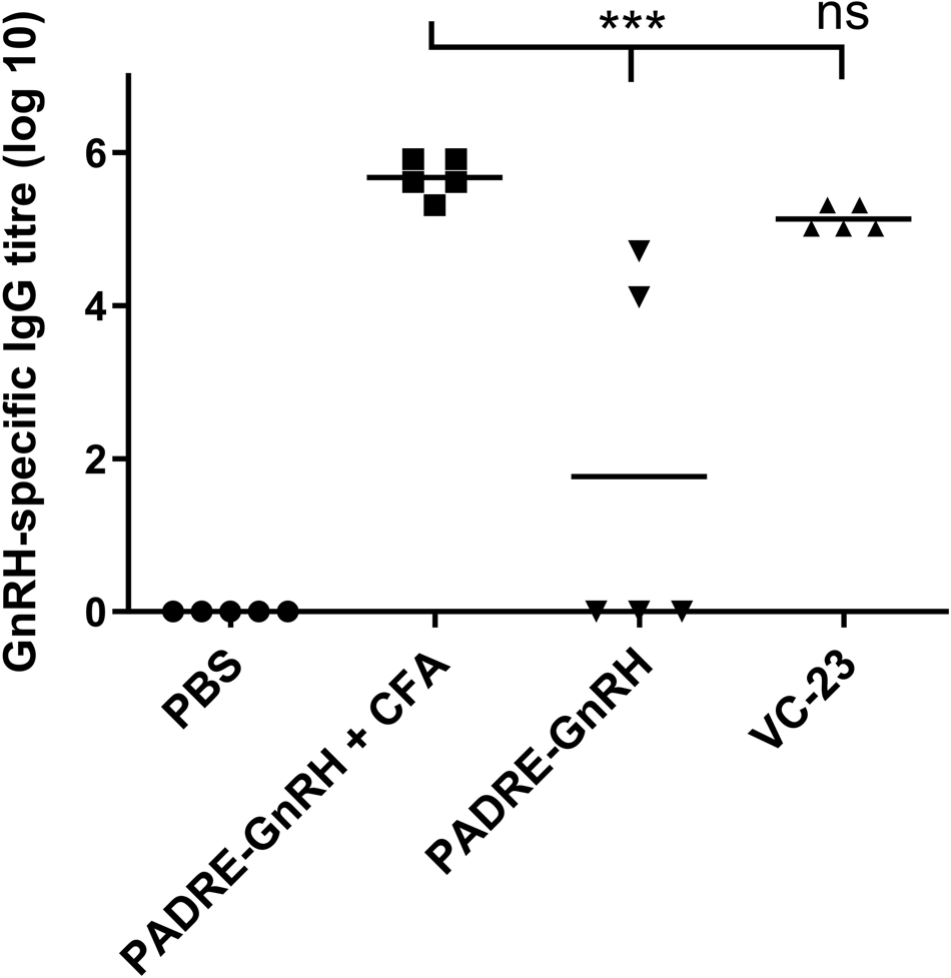
GnRH-specific total IgG titres induced by subcutaneous immunisation. Total IgG titres (log 10) specific to GnRH on day 41, following primary immunisation and three boosts, as determined by ELISA. Mice (*n* = 5 per group) were immunised with **VC-23** (Table S1), the negative control (**PBS**) and positive controls (**PADRE-GnRH +/− CFA**). Each point represents an individual mouse with bars representing the average antigen-specific serum IgG antibody titres. Statistical analysis was performed using a one-way ANOVA followed by Tukey post-hoc test (ns, *p* > 0.05; ***, *p* < 0.001).

In addition, an IgG ELISA on day 41 showed no significant difference between the PADRE C-terminally conjugated GnRH antigen (**VC-23**) and the respective CFA positive control (Fig. 8). Notably, GnRH antigen alone (**PADRE-GnRH**) showed negligible antibody production indicating the efficacy of the adjuvant in vaccine design.

TEM to assess particle size and morphology of **VC-19** to **VC-22** was performed (Fig. S19). Here, **VC-19** and **VC-20**, both contained the same antigenic epitope NS1, produced particles of similar morphology less than 500 nm in diameter. Similarly, 88/30-derived vaccines **VC-21** and **VC-22** displayed larger particles, no greater than 1 µm in size. These TEM images confirmed that these cyclic lipopeptide-based vaccines have the ability to self-assemble and form nanoparticles allowing for the presentation of antigenic epitopes^10^. Studies have indicated that lipopeptides carrying short lipids adopt a randomly coiled secondary structure and self-assemble into nanoparticles, while longer lipids induce *β*-fold formation and protofibril production (as shown in Table 2)^32^.

### Robust Immune Responses Induced by Cyclic Lipopeptide-Based Vaccines for Protein Antigens

As antigens, proteins alone often need a little assistance to achieve the desired immune response, as evidenced by these commercial vaccines which are co-administered with an adjuvant (e.g. Bexsero^TM^, Fluad^TM^, Shingrix^TM^, Heplisav-B^TM^, and Cervarix^TM^)^4^. In this study, we adjuvanted BSA and SARS-CoV 2 RBD proteins by co-administration with our synthetic adjuvant (Table S1).

**Fig. 9:**
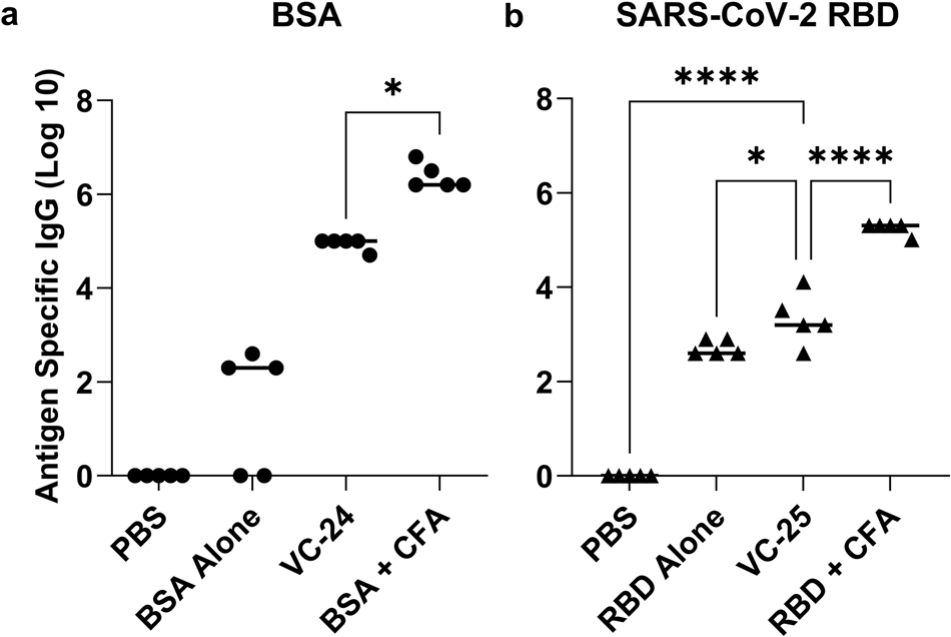
BSA- and SARS-CoV-2 RBD-specific total IgG titres induced by subcutaneous immunisation. **a** BSA-specific total IgG titres (log 10) following primary immunisation and two boosts, as determined by ELISA. **b** SARS-COV-2 RBD-specific total IgG titres (log 10) following primary immunisation and one boost, as determined by ELISA. Mice (*n* = 5 per group) were immunised with the vaccine candidates (Table S1). **VC-24** mice each received a physical mixture of BSA (20 μg), **BB4** (2.5 μg) and **BB6** (2.8 μg) in PBS (50 μL). Positive control mice each received BSA (20 μg) emulsified in CFA and PBS (1:1, total 50 μL). **VC-25** mice each received a physical mixture of SARS-CoV-2 RBD (12.5 μg), **BB4** (5.9 μg) and **BB6** (6.6 μg) in PBS (50 μL). Positive control mice each received SARS-CoV-2 RBD (12.5 μg) emulsified in CFA and PBS (1:1, total 50 μL). Each point represents an individual mouse, with bars indicating the average antigen-specific serum IgG antibody titres. Statistical analysis was performed using a one-way ANOVA followed by Tukey post-hoc test (*, *p* < 0.05; ****, *p* < 0.0001).

Not surprisingly, auto-BSA antibodies were observed when the BSA protein was administered alone (Fig. 9)^33^. Excitingly, following primary immunisation and three boosts, our adjuvant (cyclic decapeptide plus lipid; Table S1) enhanced the IgG titre for the BSA protein above these auto-BSA antibody levels in VC-24 (Fig. 9). Due to the costs associated with the SARS-CoV-2 protein, following formulation of the SARS-CoV-2 protein with adjuvant (Table S1), two immunisations were performed on day 0 and day 14 prior to euthanasia and final blood collection on day 24 of the study. Despite a reduction in the amount of total protein administered (12.5 μg/mouse), a lower number of boosts compared with previous studies using this adjuvant formulation (that is, one boost here compared with three boosts in other studies reported with this same adjuvant), and a lower amount of antigen (5 μg/plate) coated on the ELISA plate, we still observed an enhancement in IgG response compared with that of the negative (**PBS**) control and the auto-SARS-CoV-2 antibodies (Fig. 9). A pseudovirus assay validated the neutralising ability of induced antibodies. Results indicated no statistically significant difference between **VC-25** and **RBD** **+** **IFA** (positive control), and also no significant difference between RBD alone and **VC-25** (Fig. S20). TEM assessment of the BSA vaccine construct (**VC-24**; Fig. S19) indicated nanoparticle formation consistent with pervious peptide antigen-based vaccine formulations.

### Efficient Immune Responses Against Cocaine Hapten Using the Cyclic Lipopeptide Platform

There has been one clinical trial completed for an anti-cocaine vaccine, comprised of a small synthetic hapten (similar to the one shown in Table S1) conjugated to a carrier protein and co-administered with a commercial adjuvant (alum)^34–38^. This vaccine failed to induce the necessary antibody titres in phase 3 trials, and since then, a number of pre-clinical assessments have been carried out in rodent models in the quest for an anti-cocaine vaccine^18^.

**Fig. 10:**
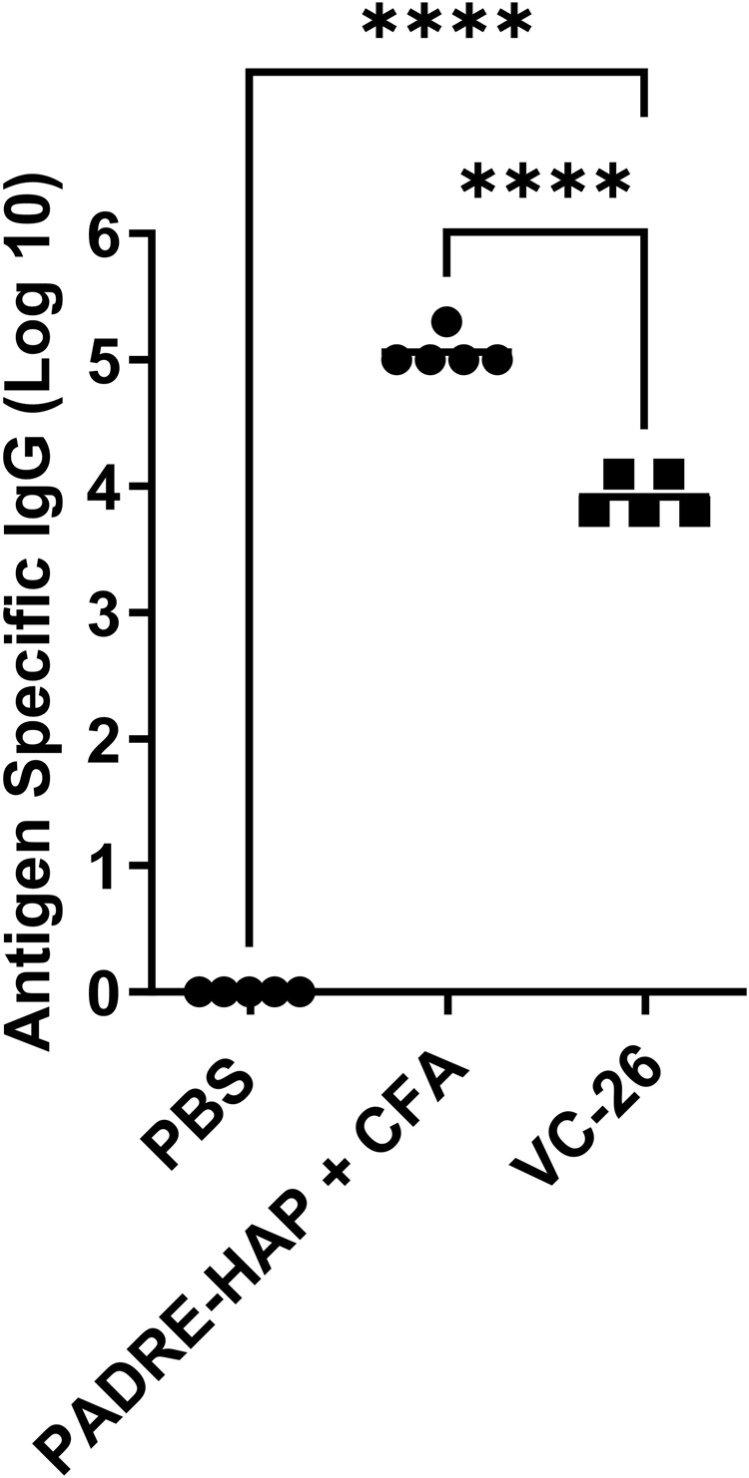
Hapten-specific total IgG titres induced by subcutaneous immunisation. Total IgG titres (log 10) specific to the hapten at day 41, following primary immunisation and three boosts, as determined by ELISA. Mice (*n* = 5 per group) were immunised with the vaccine candidates (Table S1), the negative control (**PBS**) and positive control (**PADRE-hapten [HAP]** + **CFA**). Each point represents an individual mouse with bars representing the average antigen-specific serum IgG antibody titres. Statistical analysis was performed using a one-way ANOVA followed by Tukey post-hoc test (****, *p* < 0.0001).

In this study, we adjuvanted a synthetic cocaine hapten (HAP) conjugated through a triazole linkage to the T helper peptide, PADRE (**VC-26**; Table S1)^22^. Following formulation with our adjuvant (Table S1), mice were immunised with a primary immunisation and 3 boosts before sera was collected and analysed (day 41; Fig. 10). Here, **VC-26** showed enhanced immune responses compared with the negative (**PBS**) control. TEM analysis of **VC-26** showed small aggregates (Fig. S19).

## Discussion

The cyclic lipopeptide delivery system has been previously shown to efficiently deliver vaccines and induce immunity against group A *Streptococcus*. This prompted further investigation into the underlying mechanisms of immunomodulation by cyclic peptide and KKSSC16C16. The findings suggest the potential of this system as a universal adjuvant for facilitating the delivery of various antigens, potentially offering broader application in vaccination strategies.

Here, optimisation and translation efficacy of a synthetic peptide-based adjuvant delivery system was conducted in five concurrent in vivo studies following the successful refinement of this adjuvant, from a fully-conjugated single-unit system to a physically-mixed adjuvant platform (Fig. 1)^9^. Optimisation of the vaccine schedule was first conducted, adjusting the adjuvant delivery system to peptide antigen ratio, followed then by an assessment of translational efficacy of this physically-mixed synthetic peptide-based adjuvant in peptides, proteins and a small hapten vaccine model.

An adjuvant ratio of eight times that of the original vaccine (**VC-13**) gave enhanced immunological results and opsonic ability, leading to a reduction in the number of immunisations needed without affecting disease protection. The biodistribution study underscored the notable and direct lymph nodes targeting ability of cyclic peptide. Moreover, the addition of cyclic lipopeptide improved the overall organ targeting of free peptide. In vitro cellular studies revealed the cyclic lipopeptide augmented DC uptake compared with peptide alone and demonstrated significantly enhanced DC activation through specific targeting of TLR2 and TLR4 on the DC surface.

The adjuvant (cyclic peptide and lipid) physically mixed wsith different peptide antigens, including NS1 and 88/30 from GAS and the GnRH peptide, enhanced the immune response to that of the antigen plus commercial adjuvant (CFA) following a primary immunisation and three boosts, and/or antigen alone.

The synthetic adjuvant successfully enhanced the vaccine immune response of both the BSA and SARS-CoV-2 RBD protein antigens, when compared with the protein alone (or protein plus commercial adjuvant, CFA). These are very promising results for this synthetic adjuvant, where tailoring of the adjuvant-to-protein ratio, and vaccine scheduled may provide an even better response in future studies.

Our synthetic adjuvant moderately enhanced a cocaine hapten-T helper complex in a murine model, providing scope for future research development of this adjuvant in vaccines of small molecule haptens.

This multi-part structure-activity study highlighted the versatility of the physically mixed cyclic decapeptide and lipid adjuvant as part of a three-component vaccine against peptide, protein and small molecule hapten. Further, this study revealed that an increase in adjuvant to antigen ratio allowed for a reduction in the number of immunisations without compromising immune response or opsonic activity. Promising and significant adjuvant activity was also observed when co-administered with different peptide antigens, proteins and a small hapten, providing moderate-strong antibody titres compare with antigens alone. The results presented here support further investigation of these building blocks as potential vaccine adjuvants for other peptide, protein and synthetic hapten antigens, as well as scope for refinement of the vaccine dose leading to universal adjuvant translation.

## Methods

### Synthesis of vaccine building blocks

**PADRE-J8** (**BB3**, N_3_-AKFVAAWTLKAAA-QAEDKVKQSREAKKQVEKALKQLEDKVQ), lipid (**BB4**, NH_2_-**KKSS-C16-C16**-CONH_2_) and cyclic decapeptide (**BB6**, -AK_(alkyne)_APGKK_(alkyne)_APG-) were synthesised and purified as previously reported (Fig. S1-S3, respectively)^9–11^. **NS1-PADRE** (N_3_-RVTTRSQAQDAAGLKEKADC-AKFVAAWTLKAAA; Fig. S4), **PADRE-NS1** (N_3_-AKFVAAWTLKAAA-RVTTRSQAQDAAGLKEKADC; Fig. S5), **88/30-PADRE** (N_3_-DNGKAIYERARERALQELGPC-AKFVAAWTLKAAA; Fig. S6), **PADRE-88/30** (N_3_-AKFVAAWTLKAAA-DNGKAIYERARERALQELGPC; ; Fig. S7) and **GnRH-PADRE** (EHWSYGLRPG-AKFVAAWTLKAAA; Fig. S8) were synthesised on Rink amide MBHA resin (100–200 mesh; resin substitution: 0.65 mmol/g; Novabiochem, L¨aufelfingen, Switzerland) as per our previous method^39^. Lipid-peptide synthesis (NH_2_-**KKSS-C16-C16**-CONH_2_ Fig. S2) was achieved on Rink amide MBHA resin using standard Fmoc SPPS protocol, where C16 represents the 16-length carbon chain lipoamino acid, DDe-C16, synthetically prepared in solution prior to resin coupling^40^. Linear confirmation of the cyclic peptides, **BB6** (-AK_(alkyne)_APGKK_(alkyne)_APG-) were synthesised on 2-chlorotrityl chloride resin 1% DVB (100–200 mesh; resin substitution: 1.14 mmol/g; Novabiochem, L¨aufelfingen, Switzerland) allowing for a free carboxyl group at the C-terminus as previously reported^9^. Solution-phase head-to-tail cyclisation was achieved using a coupling mixture of 1-[Bis(dimethylamino)methylene]-1H-1,2,3-triazolo[4,5-b]pyridinium 3-oxid hexafluoro phosphate (HATU) and diisopropylethylamine (DIPEA) in N,N-dimethylformamide (DMF) in a good yield^9^.

**Cy5.5-PADRE-J8 [Cy5.5-BB3], (Cy5.5)**_**2**_**-KKKSS-C16-C16 [Cy5.5-BB4] and (Cy5.5)**_**2**_**-cyclic decapeptide [Cy5.5-BB6]** were synthesised by a copper (I) catalysd alkyne-azide 1,3-dipolar cycloaddition click reaction by conjugation of **BB3,** **BB4** and **BB6** respectively with Cy5.5-alkyne (Lumiprobe, Hunt Valley, Maryland, USA) as described in the literature (Figs. S10–12)^21^. Briefly, Cy5.5-alkyne (0.635 mg, 0.001 mmol, 1.2 eq.) was dissolved in dimethyl sulfoxide (DMSO, 10 mM, Sigma-Aldrich) and added to a solution of **N**_**3**_**-PADRE-J8** (4 mg, 0.00085 mmol, 1 eq.) in a MeOH/H_2_O solution (5 mL, 50% v/v). After mixing the two solutions together, CuSO_4_ (8.8 eq.) and NaAsc (17.6 eq.) dissolved in Milli-Q water were added and the reaction was stirred for 2 hr at 50 °C. Analytical (reverse phase) RP-HPLC (Shimadzu, Kyoto, Japan) and electrospray ionisation mass spectrometry (ESI-MS, Concord, ON, Canada) monitored the reaction before two drops of trifluoroacetic acid (TFA) was added to the reaction mixture until the suspension disappeared. **Cy5.5-BB3 and Cy5.5-BB6** were obtained and purified by preparative RP-HPLC (C18 column, 20 mL/min) with a gradient of 40% solvent B to 70% solvent B to produce a blue solid. **Cy5.5-BB4** was obtained and purified by preparative RP-HPLC (C4 column, 10 mL/min) with a gradient of 70% solvent B to 100% solvent B to produce a blue solid.

The synthesis of cocaine **PADRE-hapten [HAP]** was undertaken as per Madge et al., and was functionalised with an azide group to enable conjugation to a T helper (PADRE) peptide alkyne through azide-alkyne cycloaddition in solution (Fig. S9)^22^.

Purification was performed using semi-preparative RP-HPLC on a Shimadzu (Kyoto, Japan) liquid chromatography system (CBM-20A controller, FRC-10A fraction collector, LC-20AT pump, SIL-10A auto injector, SPD-20A UV/Vis, set to a wavelength of 214 nm) with either a Grace Alltima C18 column (5 μm, 22 mm diameter, 250 mm length, flow rate: 20 mL/min) or Grace Vydac HPLC 214 C4 column (5 μm, 22 mm diameter, 250 mm length, flow rate: 10 mL/min) in a gradient of solvent A (Milli-Q water with 0.1% TFA) and solvent B (90% MeCN in Milli-Q water with 0.1% TFA). *>*95% pure final components were measured by analytical RP-HPLC liquid chromatography system (DGU-20A5 degasser, LC-20AB pump, SIL-20AC HT auto sampler, SPD-M10A VP detector, set to a wavelength of 214 nm) with either a Grace Vydac 218TP C18 column (5 μm, 4.6 mm diameter, 250 mm length) or Grace Vydac 214TP C4 column (5 μm, 4.6 mm diameter, 250 mm length) at a 1 mL/min flow rate in a gradient of solvent A and solvent B.

ESI-MS was performed on the Perkin-Elmer-Sciex API3000 triple quadrupole mass spectrometer with a flow of the mixture of solvent C (Milli-Q water with 0.1% AcOH) and solvent D (90% MeCN in Milli-Q water with 0.1% AcOH) (1:1) at a constant rate of 0.5 mL/min, and MS data were collected and analysed with Analyst 1.4 software (Applied Biosystems/ MDS Sciex, Toronto, Canada). All the samples were lyophilised on a Christ TQP501 freeze dryer.

### In vivo immunological assessment

Five independent structure-activity vaccine studies were carried out consecutively on different antigens (Fig. 1). We initially assessed the ratio of adjuvant (lipid [**BB4**] and cyclic decapeptide **[BB6]**) to J8 GAS B cell antigen as a physically mixed vaccine. We then assessed the efficacy of the adjuvant in the presence of different GAS B cell epitopes (NS1 and 88/30) conjugated to the N- and C-terminus of the T helper epitope PADRE, and the efficacy of the adjuvant in the presence of the GnRH peptide conjugated to the C-terminus of the T helper epitope PADRE. Lastly, we investigated the adjuvant properties of the lipid and cyclic decapeptide towards proteins (BSA and SARS-CoV-2 RBD) and a small molecule hapten antigen, respectively.

All animal study procedures and protocols were approved by the University of Queensland Animal Ethics Committee (AEC) with AEC approval (SCMB/AIBN/069/017) in accordance with National Health and Medical Research Council (NHMRC) of Australia. C57BL/6 mice were obtained from the Animal Resource Centre (Perth, Western Australia) and housed at the AIBN UQBR facility (Brisbane, QLD, Australia).

C57BL/6 mice (female, 4–6 weeks old, 5 mice/group) were acclimatised for 7 days before the experiment. All mice received four immunisation doses on days 0, 21, 28, and 35. Each mouse was subcutaneously immunised at the tail base with 30 μg of antigen or an antigen-normalised dose of varying composition of the physical mixture (lipid, cyclic peptide and antigen) dissolved in phosphate-buffered saline (PBS; 50 μL). Negative control mice each received PBS (50 μL). Positive control mice each received PADRE-J8 (30 μg) emulsified in CFA and PBS (1:1, total 50 μL) for the primary immunisation followed by three boosts of PADRE-J8 (30 μg) in PBS (50 μL). Serum was collected on days 1, 20, 27, and 34 to determine the level of antigen-specific immunoglobulin G (IgG) antibodies. Blood was collected by tail bleed. On day 41, the mice were euthanised with a CO_2_ inhalation chamber and the neat blood was collected by cardiac bleed. Serum was collected following centrifugation (4000 rpm, 20 min) and the supernatant was kept at -80 °C for further analysis.

### Determination of antigen-specific antigen titres by ELISA

An enzyme-linked immunosorbent assay (ELISA) was performed to measure J8-specific IgG according to the literature^9–11^. 96-Well plates were coated with a solution of the antigen (peptide, protein or hapten heterologous peptide conjugate [HAP-ALKQLEDKVQ]) in carbonate coating buffer (5 μg peptide/plate, 100 μL/well) and incubated for 90 min at 37 °C^22^. Following plate washing (PBS-tween-20 buffer), wells were treated overnight with a blocking solution (150 μL/well) made up of 5% skim milk in PBS with 0.05% Tween-20. Following plate washing, sera (diluted 1:100 in 0.5% skim milk/PBS-tween-20 buffer) was serially diluted 1:2 down the plate and incubated (90 min, 37 °C). To detect mice antigen-specific antibodies, horseradish peroxidase (HRP)-conjugated goat anti-mouse total IgG antibody (using a dilution of 1:3000; Bio-Rad Laboratories, Gladesville, NSW, Australia), IgG1 secondary antibody (using a dilution of 1:6000; Invitrogen, Thermo Fisher Scientific, Waltham, MA, USA) and IgG2c antibody (using a dilution of 1: 10000; Abcam, Cambridge, UK) were added to the washed wells and incubated (90 min, 37 °C). Following washing, plates were treated with o-phenylenediamine dihydrochloride (OPD) solution (100 μL/well) and the absorbance was measured at 450 nm by a SpectraMAX 250 plate reader (Molecular Devices, San Jose, CA, USA). Antibody titres (IgG, IgG1, and IgG2c) are defined as the lowest dilution with an optical density more than three times the standard deviation greater than the average value of the optical density of the naïve sera. Statistical significances were calculated with one-way ANOVA followed by Tukey’s post-hoc test with GraphPad Prism 10 software.

### Opsonisation assay

Opsonisation assay was performed as per a previously published protocol^9–11^. Briefly, two GAS clinical isolates (ACM-2727 [Royal Brisbane Hospital]; D3840 [nasopharynx swab]) provided by Princess Alexandra hospital were prepared by steaking into Todd-Hewitt broth (THB) agar plates (supplemented with 5% yeast extract) and allowed to culture (37 °C, 24 h). A single colony from each isolate was transferred into 5 mL THB with 5% yeast extract for another 24 h incubation (37 °C) to get almost 4.6 × 10^7^ colony forming units (CFU)/mL. The cultivate was serially diluted to 10^-2^ CFU/mL in PBS with an aliquot (10 μL) that was mixed with inactivated day 27 tail sera (diluted 1:10 in PBS, 10 μL in total, water bath 50 °C, 10 min) or day 41 cardiac sera (10 μL, water bath 50 °C, 10 min) and horse blood (80 μL). The assay was performed in duplicate on three independent cultures. Strains were cultured in a 96-well THB agar plate with sera (37 °C, 3 h) where an aliquot (10 μL) extracted from culture material was added into the THB plates agar with 5% yeast extract and 5% horse blood. Plates were incubated (37 °C, 24 h) and analysed based on CFU enumeration to obtain the bacterial survival rate. The opsonic activity of the antibodies mean percentage reduction in mean CFU was calculated.

### Transmission electron microscopy

Vaccine compounds (0.1 mg/mL in PBS) were applied to glow-discharged carbon-coated copper 200 mesh grids (Ted Pella) and negative-stained with 2% uranyl acetate. Particle image was captured using a JEM-1010 TEM (HT7700 Exalens, Hitachi Ltd., Japan) operated at 80 kV.

### Biodistribution assay

C57BL/6 mice were subcutaneously immunised with a single dose of PBS (50 μL/mouse), **Cy5.5-BB3** (28 μg/mouse in 50 μL PBS), **Cy5.5-BB4,** **Cy5.5-BB6** or **Cy5.5-VC-13** (a physical mixture of **Cy5.5-BB3,** **BB4** and **BB6** with an antigen-normalised dose of **VC-13** in 50 μL PBS) and were sacrificed in groups of three at four-time points (4, 8, 12, and 24 h). Following euthanasia by CO_2_ inhalation, mice were dissected and organs (heart, lungs, kidneys, spleen, lymph nodes, and liver) were imaged on a PerkinElmer IVIS Lumina X5 for fluorescence intensity (676 nm excitation, 705 nm emission). Fluorescence intensity was recorded as radiant efficiency and reported as percent fluorescence.

### Dendritic cell maturation assay

DC2.4 Cells (Thermo Fisher Scientific, Waltham, MA, USA) from C57BL/6 mice were cultured in RPMI-1640 medium supplemented with FBS (10% v/v), HEPES buffer (2.5% v/v), L-glutamine (1% v/v), NEAA (1% v/v), PSG (1% v/v) and 2-mercaptomethanol (0.00054% v/v) at 37 °C in a CO_2_ (5%) incubator. DC2.4 cells were seeded, treated with vaccine candidates, incubated, collected, and stained successively as previously reported^26^. Briefly, DC2.4 cells (4.5 × 10^5^/well) were seeded in a 48-well plate for 24 h at 37 °C. Samples (PBS: negative control; LPS (Thermo Fisher Scientific, Waltham, MA, USA) and Pam2 CSK4 (Sapphire Bioscience Pty Limited, Redfern, Australia): positive control; cyclic decapeptide [**BB6**], KKSS-C16-C16 **[BB4]**, PADRE-J8 **[BB3]**, and **VC-13**) were added to each well at 20 μM in triplicates with RPMI-1640 media with supplementary and the cells were allowed for activation for 24 h at 37 °C. Disassociation of the cells from the plate was achieved by trypsin (100 μL/well) for 5 min, prior to transferring the cells to microcentrifuge tubes containing 100 μL supplemented RPMI-1640 media. The cells were then centrifuged at 1,700 rpm for 5 min and washed with PBS. FcR blocking solution (TruStain^TM^ FcX 1:200 diluted in PBS; BioLegend, San Diego, United States) was added to re-suspend all cells for 25 min on ice in the dark. After incubation, the cells were centrifuged and re-washed with PBS before re-suspending in an antibody cocktail of PE/Cyanine 5 anti-mouse CD40, APC anti-mouse I-A/I-E and Zombie Aqua^TM^ dye (each diluted 1:200 in PBS; BioLegend). The cells were incubated for 25 min on ice in the dark, and the centrifugation steps were repeated to wash the cells with PBS. Paraformaldehyde (PFA) solution (4% in PBS) was used to fix the cells for 15 min at RT. The cells were washed with PBS and re-suspended in PBS prior to analysis by flow cytometry. Fluorescence minus one (FMO) controls were prepared by staining the cells with minus one fluorescent antibody in the antibody cocktail. Single stained control was prepared by incubating anti-rat and anti-Hamster Igκ/Negative control compensation beads with each antibody, respectively. Live/dead control was prepared by microwaving the cells 5 seconds for three times, followed by fixation. Unstained control was prepared by skipping the staining procedure for fixation.

### Dendritic cell uptake assay

DC2.4 cells (4.5 × 10^5^/well) were seeded in a 48-well plate for 24 h at 37 °C. Samples (PBS: negative control; **Cy5.5-BB3, Cy5.5-BB4, Cy5.5-BB6** and **Cy5.5-VC-13**) were added to each well at 0.5 μM (5×) of final concentration as comparison in triplicates with supplemented RPMI-1640 media, as mentioned above, and the cells were incubated for 4 h prior to cell collection by trypsin. Collected cells centrifuged at 1700 rpm for 5 min. After removing the supernatant, the cells were washed with PBS and centrifuged. Zombie Aqua^TM^ dye (1:200 diluted in PBS) was added to re-suspend the cells on ice in the dark for 25 min prior to centrifuging to remove the supernatant. After washing cells with PBS, the cells were fixed by PFA (4% in PBS) for 15 min at RT. Cells were rewashed with PBS, re-suspended in PBS and analysed by flow cytometry. Single stained controls were prepared by incubating the cells with 25× Cy5.5-labeled compounds (2.5 μM) under the same condition, without staining the cells by Zombie Aqua^TM^ dye. Live/dead and unstained controls were prepared as described above.

### Flow cytometry analysis

The acquisition of flow cytometry events was achieved using a BD LSRFortessa^TM^ X-20 Cell Analyzer with BD FACSDiva software (BD Biosciences, Franklin Lakes, USA). The compensation set-up was conducted using beads/single stained cells. Data for full-stained and FMO samples were acquired after compensation, and at least 10,000 events were recorded for each sample in triplicate. Finally, the data was exported as FCS files and analysed using FlowJoTM v10.8 software (BD Life Sciences).

## Supplementary information

Supporting Information

## Data Availability

The datasets generated and analysed during this study are presented within this manuscript. Additional data supporting the findings are available from the corresponding author upon reasonable request.
