# Supplementary material for: A synthetic cyclic peptide for promoting antigen presentation and immune activation
Source: NPJ Vaccines. 2025 Jan 15;10:9. doi: 10.1038/s41541-024-01050-4 (PMC11733015; doi:10.1038/s41541-024-01050-4)
Supplement: Supplementary file 1 — Supporting Information [file 41541_2024_1050_MOESM1_ESM.pdf]

Supplementary Information

A synthetic cyclic peptide for promoting antigen presentation and immune activation

Jiahui Zhang[1], Harrison Y.R. Madge[1], Asmaa Mahmoud[1], Lantian Lu[1,2], Wanyi Wang[1], Wenbin Huang[1], Prashamsa Koirala[1], Jazmina Gonzalez Cruz[2], Wei Yang Kong[2], Sahra Bashiri[1], Ahmed O. Shalash[1], Waleed M. Hussein[3], Zeinab G. Khalil[3], James W. Wells[2], Istvan Toth[1,3,4] and Rachel J. Stephenson[1,]*

[1]School of Chemistry and Molecular Biosciences, The University of Queensland, Brisbane QLD 4072, Australia.

[2]Faculty of Medicine, Frazer Institute, The University of Queensland, Brisbane, QLD 4102, Australia.

[3]Institute for Molecular Bioscience, The University of Queensland, Brisbane, QLD 4072, Australia.

[4]School of Pharmacy, The University of Queensland, Brisbane, QLD 4102, Australia.

*Corresponding Author:

Dr Rachel Stephenson

The School of Chemistry and Molecular Biosciences

The University of Queensland

Brisbane

Australia

Phone: (617) 3346 9893

Fax: (617) 3365 4273

Email: r.stephenson@uq.edu.au

Table of Contents

Fig. S1 Mass spectra and RP-HPLC trace for PADRE-J8..3

Fig. S2 Mass spectra and RP-HPLC trace for NH_2-KKSS-C16-C16-$CONH_2$..........................3

Fig. S3 Mass spectra and RP-HPLC trace for cyclic decapeptide...4

Fig. S4 Mass spectra and RP-HPLC trace for NS1-PADRE...4

Fig. S5 Mass spectra and RP-HPLC trace for PADRE-NS1...5

Fig. S6 Mass spectra and RP-HPLC trace for 88/30-PADRE...5

Fig. S7 Mass spectra and RP-HPLC trace for PADRE-88/30...6

Fig. S8 Mass spectra and RP-HPLC trace for GnRH-PADRE..6

Fig. S9 Mass spectra and RP-HPLC trace for PADRE-hapten. 7

Fig. S10 Mass spectra and RP-HPLC trace for Cy5.5-PADRE-J8..7

Fig. S11 Mass spectra and RP-HPLC trace for Cy5.5-KKSSC16C16.....................................8

Fig. S12 Mass spectra and RP-HPLC trace for Cy5.5-cyclic peptide. 8

Fig. S13 J8-specific total IgG titres (log 10) measured at day 34 (A) and day 41 (B). 9

Fig. S14 Biodistribution and organ-specific fluorescence imaging of Cy5.5-labeled vaccine components post-immunisation. 11

Fig. S15 Gating strategy and expression analysis of activation markers in DC2.4 cells.........11

Fig. S16 Expression histograms of CD40 and MHC-II expression in DC2.4 cells................12

Fig. S17 Representative histograms of CD86 and CD80 expression in DC2.4 cells. 12

Fig. S18 Representative histograms showing the expression of toll-like receptors 2 and 4 in DC2.4 cells...13

Table S1. Schematic illustration of vaccine formulations. 14

Fig. S19 Transmission electron microscopy (TEM) of VC-19, VC-20, VC-21, VC-22, VC-23, VC-24 and VC-26 (0.1 mg/mL)..15

Fig. S20 Pseudovirus neutralization assay..16

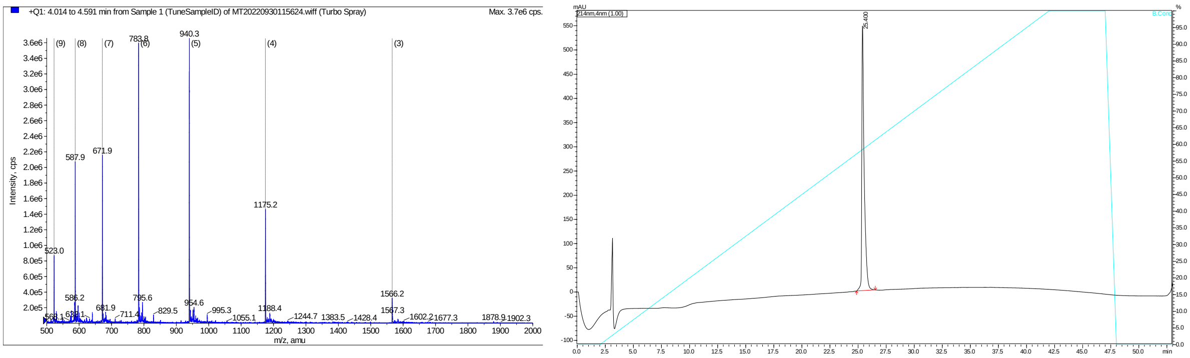

Fig. S1 Mass spectra and RP-HPLC trace for PADRE-J8.

PADRE-J8 sequence: N_3-AKFVAAWTLKAAA-QAEDKVKQSREAKKQVEKALKQLEDKVQ. Yield: 25%. Rt = 25.4 min (C18 column, 0-100% solvent B, 42 min). Molecular weight ($C_{207}H_{347}N_{63}O_{61}$): 4694.43 g/mol. ESI-MS: $[M + 3H]^{+3}$ m/z 1566.2 (calcd: 1565.8), $[M + 4H]^{+4}$ m/z 1175.2 (calcd: 1174.6), $[M + 5H]^{+5}$ m/z 940.3 (calcd: 939.9), $[M + 6H]^{+6}$ m/z 783.8 (calcd: 783.4), $[M + 7H]^{+7}$ m/z 671.9 (calcd: 671.6), $[M + 8H]^{+8}$ m/z 587.9 (calcd: 587.8), $[M + 9H]^{+9}$ m/z 523.0 (calcd: 522.6).

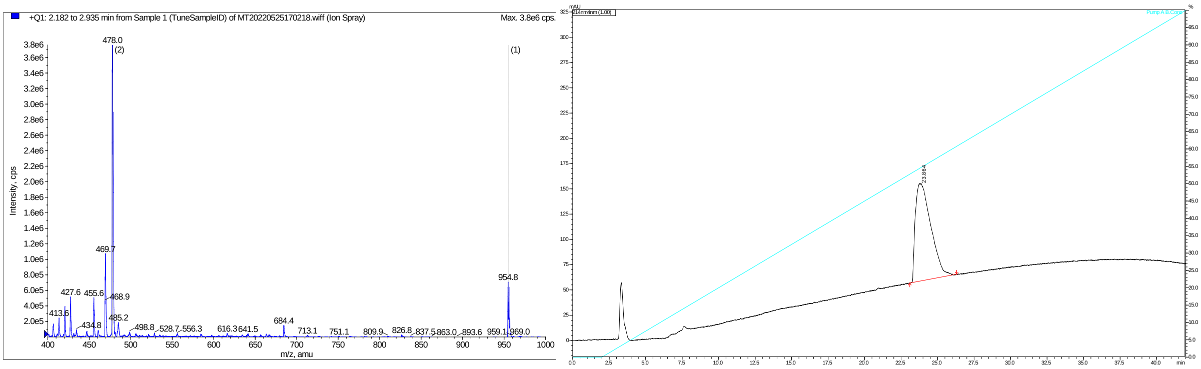

Fig. S2 Mass spectra and RP-HPLC trace for NH_2-KKSS-C16-C16-$CONH_2$.

Yield: 50%. R_t = 23.8 min (C4 column, 0-100% solvent B, 42 min). Molecular weight ($C_{50}H_{99}N_9O_8$): 954.4 g/mol. ESI-MS: $[M + 1H]^{+1}$ m/z 954.8 (calcd: 955.4), $[M + 2H]^{+2}$ m/z 477.9 (calcd: 478.2).

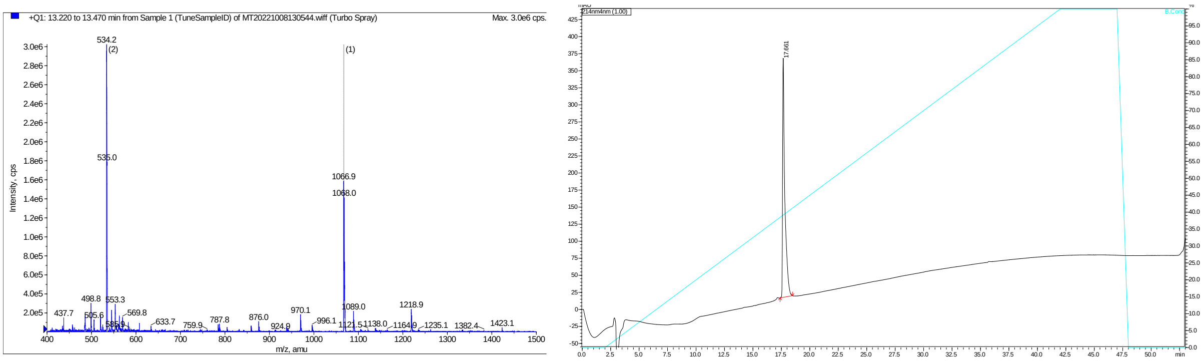

Fig. S3 Mass spectra and RP-HPLC trace for cyclic decapeptide.

Cyclic peptide BB6 sequence: -AK$_{(alkyne)}$APGKK$_{(alkyne)}$APG-. Yield: 15%. Rt = 17.7 min (C18 column, 0-100% solvent B, 42 min). Molecular weight ($C_{51}H_{79}N_{13}O_{12}$): 1066.27 g/mol. ESI-MS: [M + 1H]$^{+1}$ m/z 1066.9 (calcd: 1067.3), [M + 2H]$^{+2}$ m/z 534.2 (calcd: 534.1).

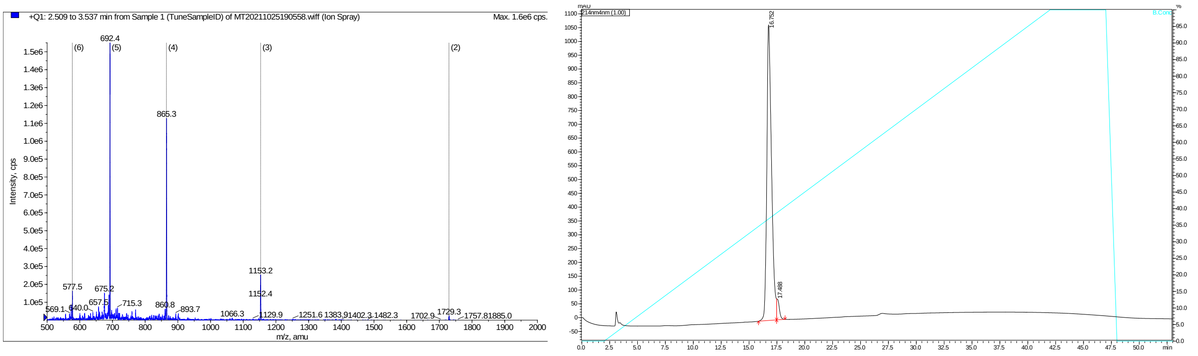

Fig. S4 Mass spectra and RP-HPLC trace for NS1-PADRE.

NS1-PADRE sequence: N_3-RVTTRSQAQDAAGLKEKADC-AKFVAAWTLKAAA. Yield: 25%. Rt = 16.7 min (C18 column, 0-100% solvent B, 42 min). Molecular weight ($C_{150}H_{247}N_{49}O_{45}$): 3456.92 g/mol. ESI-MS: [M + 2H]$^{+2}$ m/z 1729.3 (calcd: 1729.5), [M + 3H]$^{+3}$ m/z 1153.2 (calcd: 1153.3), [M + 4H]$^{+4}$ m/z 865.3 (calcd: 865.2), [M + 5H]$^{+5}$ m/z 692.4 (calcd: 692.4), [M + 6H]$^{+6}$ m/z 577.5 (calcd: 577.2).

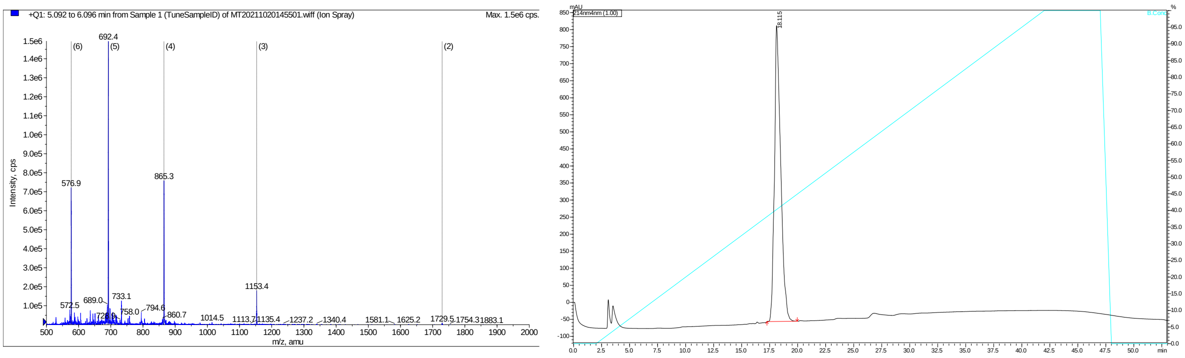

Fig. S5 Mass spectra and RP-HPLC trace for PADRE-NS1.

PADRE-NS1 sequence: N_3-AKFVAAWTLKAAA-RVTTRSQAQDAAGLKEKADC. Yield: 25%. Rt = 18.1 min (C18 column, 0-100% solvent B, 42 min). Molecular weight ($C_{150}H_{247}N_{49}O_{45}$): 3456.92 g/mol. ESI-MS: $[M + 2H]^{+2}$ m/z 1729.5 (calcd: 1729.5), $[M + 3H]^{+3}$ m/z 1153.4 (calcd: 1153.3), $[M + 4H]^{+4}$ m/z 865.3 (calcd: 865.2), $[M + 5H]^{+5}$ m/z 692.4 (calcd: 692.4), $[M + 6H]^{+6}$ m/z 576.9 (calcd: 577.2).

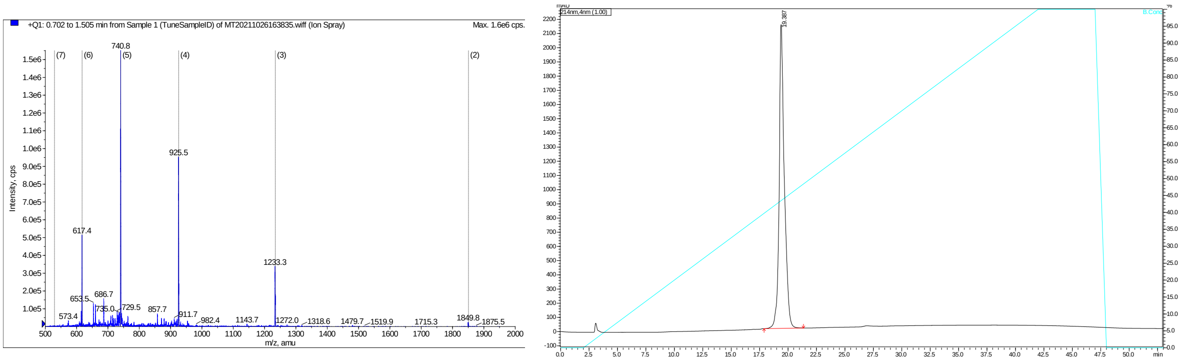

Fig. S6 Mass spectra and RP-HPLC trace for 88/30-PADRE.

88/30-PADRE sequence: N_3-DNGKAIYERARERALQELGPC-AKFVAAWTLKAAA. Yield: 25%. Rt = 19.4 min (C18 column, 0-100% solvent B, 42 min). Molecular weight ($C_{164}H_{262}N_{52}O_{46}$): 3698.22 g/mol. ESI-MS: $[M + 2H]^{+2}$ m/z 1849.8 (calcd: 1850.1), $[M + 3H]^{+3}$ m/z 1233.3 (calcd: 1233.7), $[M + 4H]^{+4}$ m/z 925.5 (calcd: 925.6), $[M + 5H]^{+5}$ m/z 740.8 (calcd: 740.6), $[M + 6H]^{+6}$ m/z 617.4 (calcd: 617.4).

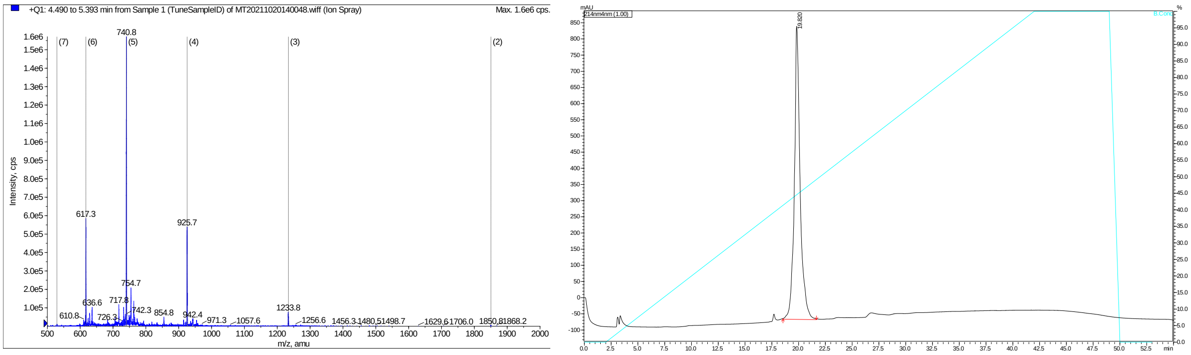

Fig. S7 Mass spectra and RP-HPLC trace for PADRE-88/30.

PADRE-88/30 sequence: N_3-AKFVAAWTLKAAA-DNGKAIYERARERALQELGPC. Yield: 25%. Rt = 19.8 min (C18 column, 0-100% solvent B, 42 min). Molecular weight ($C_{164}H_{262}N_{52}O_{46}$): 3698.22 g/mol. ESI-MS: [M + 2H]$^{+2}$ m/z 1850.8 (calcd: 1850.1), [M + 3H]$^{+3}$ m/z 1233.8 (calcd: 1233.7), [M + 4H]$^{+4}$ m/z 925.7 (calcd: 925.6), [M + 5H]$^{+5}$ m/z 740.8 (calcd: 740.6)), [M + 6H]$^{+6}$ m/z 617.3 (calcd: 617.4).

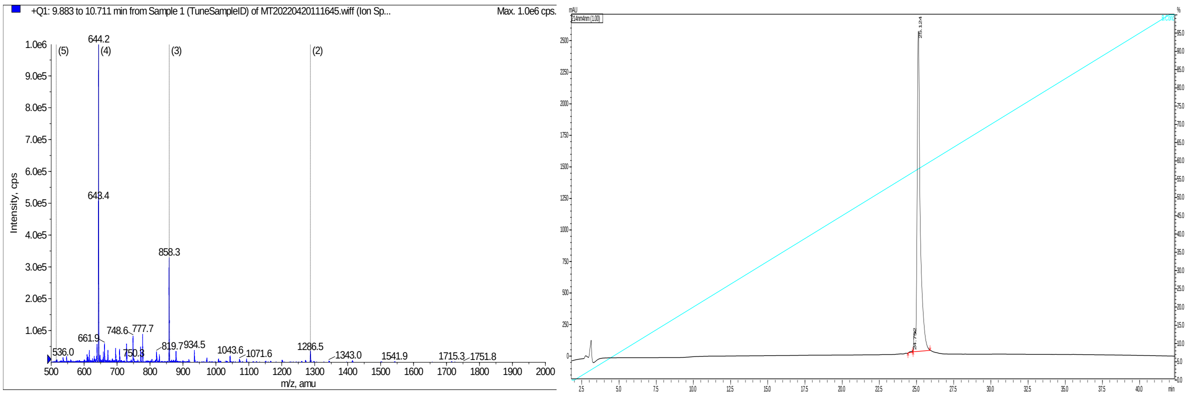

Fig. S8 Mass spectra and RP-HPLC trace for GnRH-PADRE.

GnRH-PADRE sequence: Ac-EHWSYGLRPG-AKFVAAWTLKAAA. Yield: 37%. Rt = 25.1 min (C18 column, 0-100% solvent B, 40 min). Molecular weight: 2571.9 g/mol. ESI-MS: [M + 2H]$^{+2}$ m/z 1286.5 (calcd: 1287.0), [M + 3H]$^{+3}$ m/z 858.3 (calcd: 858.3), [M + 4H]$^{+4}$ m/z 643.4 (calcd: 643.4).

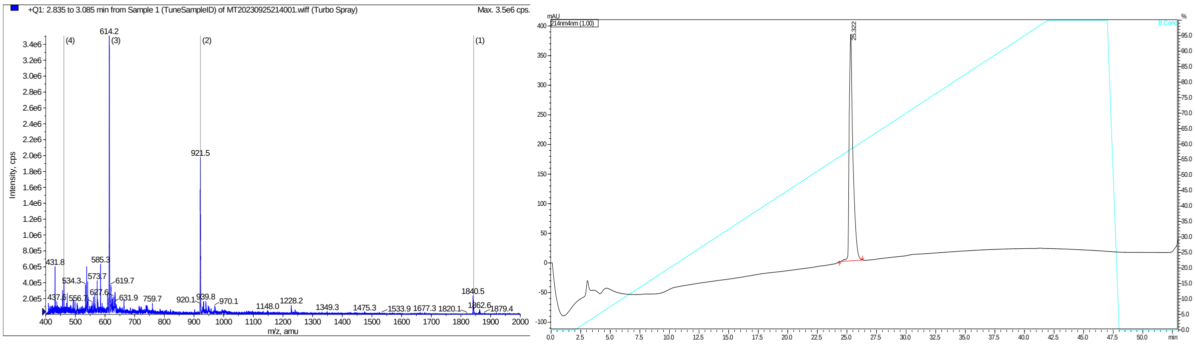

Fig. S9 Mass spectra and RP-HPLC trace for PADRE-hapten.

Yield: 60%. Rt = 25.3 min (C18 column, 0-100% solvent B, 42 min). Molecular weight ($C_{92}H_{138}N_{22}O_{18}$): 1840.25 g/mol. ESI-MS: $[M + H]^{+1}$ m/z 1840.5 (calcd: 1841.3), $[M + 2H]^{+2}$ m/z 921.5 (calcd:921.1), $[M + 3H]^{+3}$ m/z 614.2 (calcd: 614.4).

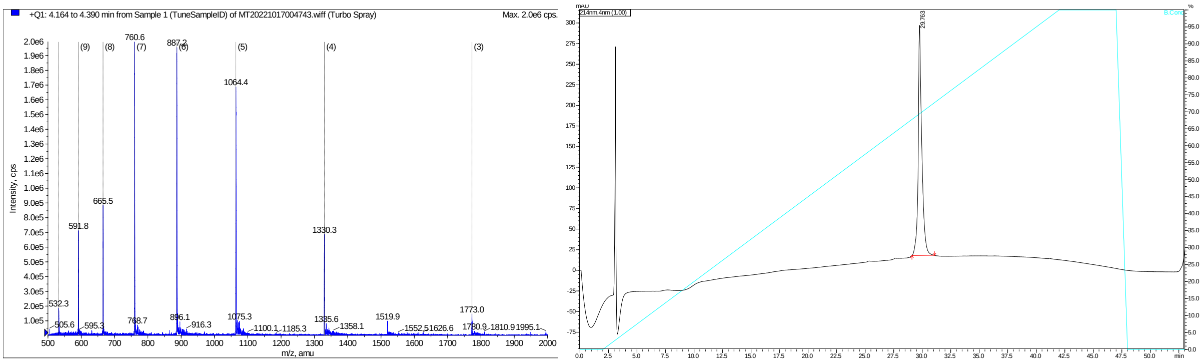

Fig. S10 Mass spectra and RP-HPLC trace for Cy5.5-PADRE-J8.

Yield: 70%. Rt = 29.7 min (C18 column, 0-100% solvent B, 42 min). Molecular weight ($C_{250}H_{393}N_{66}O_{62}$): 5315.29 g/mol. ESI-MS: $[M + 3H]^{+3}$ m/z 1773.0 (calcd: 1772.8), $[M + 4H]^{+4}$ m/z 1330.3 (calcd: 1329.8), $[M + 5H]^{+5}$ m/z 1064.4 (calcd: 1064.1), $[M + 6H]^{+6}$ m/z 887.2 (calcd: 886.9), $[M + 7H]^{+7}$ m/z 760.6 (calcd: 760.3), $[M + 8H]^{+8}$ m/z 666.5 (calcd: 665.4), $[M + 9H]^{+9}$ m/z 591.8 (calcd: 591.6), $[M + 10H]^{+10}$ m/z 532.3 (calcd: 532.5).

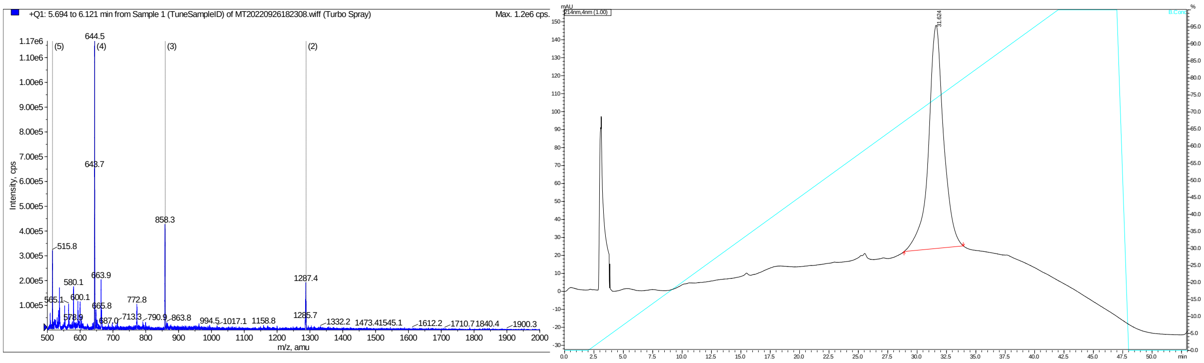

Fig. S11 Mass spectra and RP-HPLC trace for Cy5.5-KKSSC16C16.

Yield: 65%. Rt = 31.6 min (C4 column, 0-100% solvent B, 42 min). Molecular weight ($C_{152}H_{217}N_{23}O_{13}^{2+}$): 2574.55 g/mol. ESI-MS: [M + 2H]$^{+2}$ m/z 1287.4 (calcd: 1288.3), [M + 3H]$^{+3}$ m/z 858.3 (calcd:859.2), [M + 4H]$^{+4}$ m/z 644.5 (calcd: 644.6), [M + 5H]$^{+5}$ m/z 515.8 (calcd: 515.9).

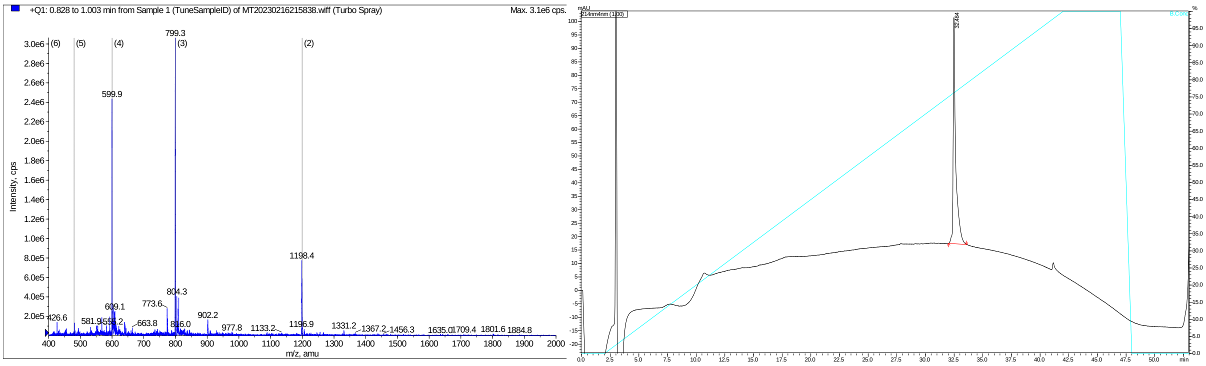

Fig. S12 Mass spectra and RP-HPLC trace for Cy5.5-cyclic peptide.

Yield: 68%. Rt = 38.5 min (C18 column, 0-100% solvent B, 42 min). Molecular weight ($C_{137}H_{177}N_{25}O_{14}^{2+}$): 2398.08 g/mol. ESI-MS: [M + 2H]$^{+2}$ m/z 1198.4 (calcd: 1200.0), [M + 3H]$^{+3}$ m/z 799.3 (calcd: 800.4), [M + 4H]$^{+4}$ m/z 599.9 (calcd: 600.5).

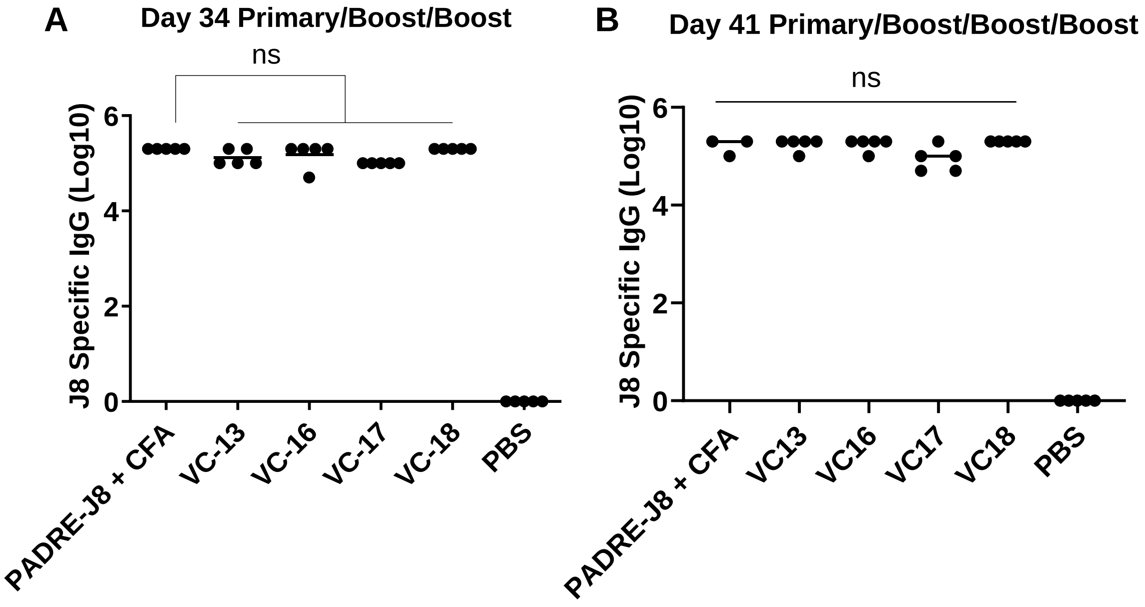

Fig. S13 J8-specific total IgG titres (log 10) measured at day 34 (A) and day 41 (B). C57BL/6 mice (n = 5 per group) were immunised subcutaneously with the indicated vaccine candidates (**Table 1**), the negative control (PBS) and positive control (PADRE-J8 + CFA). Serum IgG titres specific to J8 were determined by ELISA. Each dot represents an individual mouse, while the horizontal bars indicate the group average. Statistical analysis was performed using one-way ANOVA followed by Tukey's post-hoc test. Antibody titres against J8 are presented for each individual mouse, with group averages shown as bars (ns, $p > 0.05$).

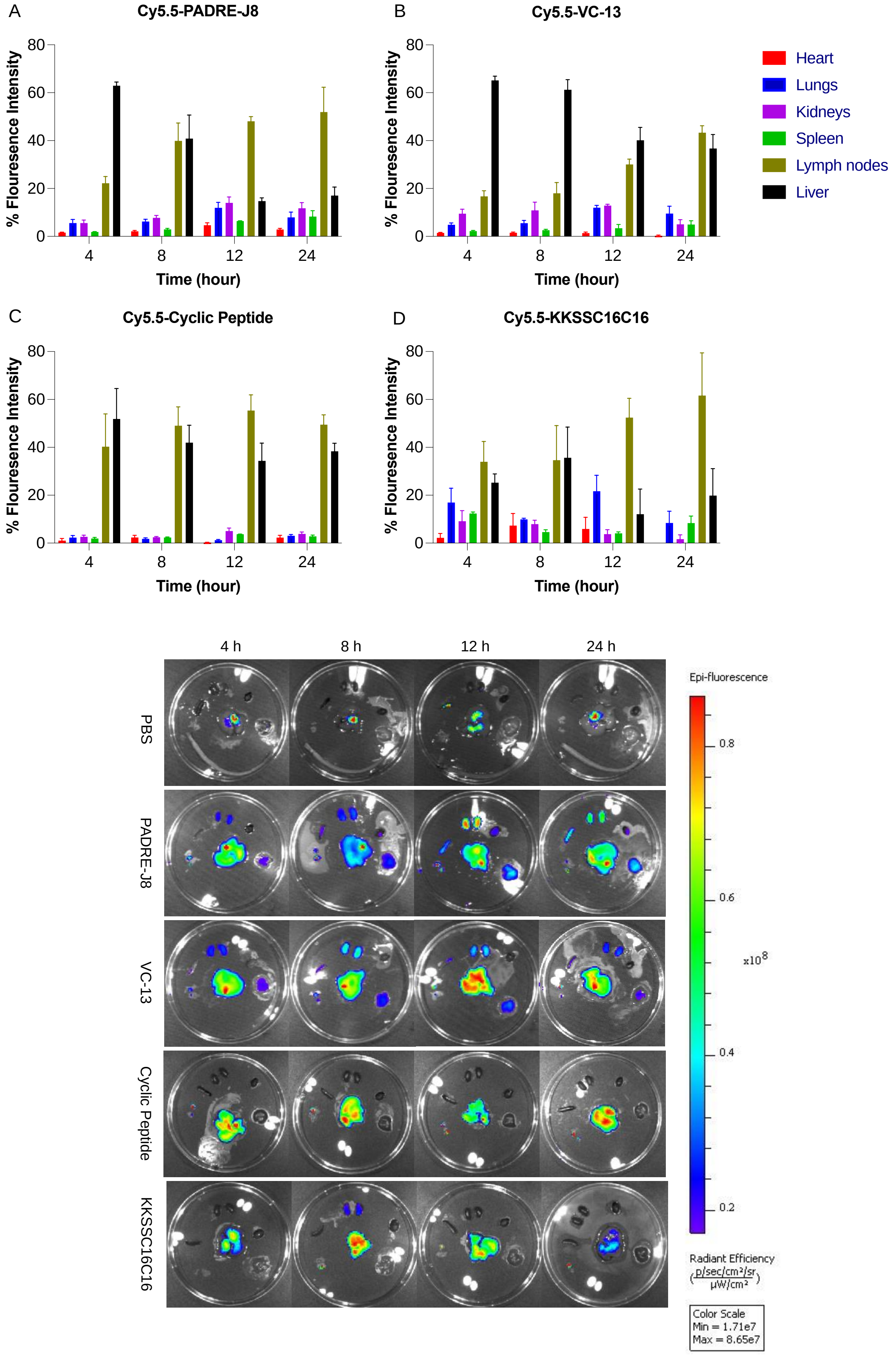
A
Cy5.5-PADRE-J8
B
Cy5.5-VC-13
C
Cy5.5-Cyclic Peptide
D
Cy5.5-KKSSC16C16
% Flouresence Intensity
Time (hour)
Heart
Lungs
Kidneys
Spleen
Lymph nodes
Liver
4 h
8 h
12 h
24 h
PBS
PADRE-J8
VC-13
Cyclic Peptide
KKSSC16C16
Epi-fluorescence
x10^8
Radiant Efficiency
(p/sec/cm²/sr / µW/cm²)
Color Scale
Min = 1.71e7
Max = 8.65e7

Fig. S14 Biodistribution and organ-specific fluorescence imaging of Cy5.5-labeled vaccine components post-immunisation.

A-D Biodistribution of Cy5.5-labeled PADRE-J8 (**A**), Cy5.5-VC-13 (**B**), Cy5.5-Cyclic Peptide (**C**) and Cy5.5-KKSSC16C16 (**D**) at 4, 8, 12 and 24 hours following a single subcutaneous immunisation (n=3 per timepoint). **E** Representative fluorescence imaging of organs (lymph nodes, spleen, kidneys, liver, heart, and lung) collected after 4, 8, 12 and 24 hours post-treatment with PBS, Cy5.5-BB3, Cy5.5-BB4, Cy5.5-BB6 and Cy5.5-VC-13.

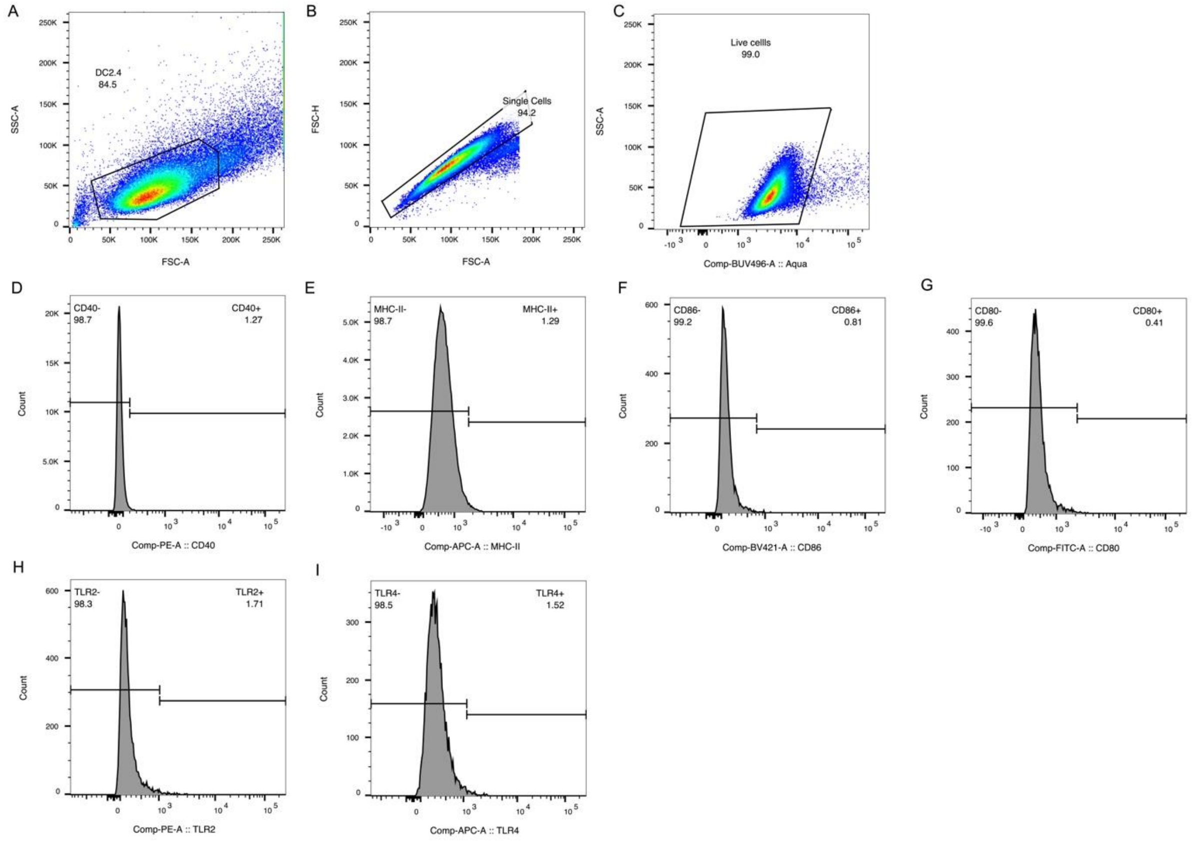

Fig. S15 Gating strategy and expression analysis of activation markers in DC2.4 cells.

A-C Representative flow cytometry gating strategy for DC2.4 cells, including the exclusion of DC2.4 cells (**A**), the selection of single cells (**B**), the gating of live cells (**C**). D-E Expression of CD40 (**D**), MHC-II (**E**), CD86 (**F**), CD80 (**G**), TLR2 (**H**) and TLR4 (**I**) on DC2.4 cells after 24 hr *in vitro* treatment. Histograms show fluorescence intensity compared to unstained controls.

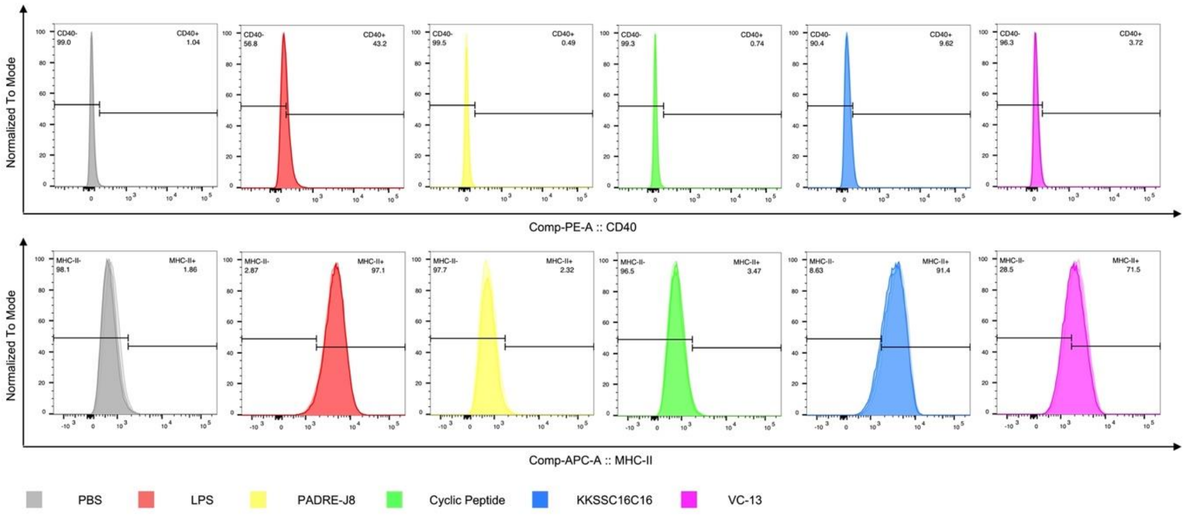

Fig. S16 Expression histograms of CD40 and MHC-II expression in DC2.4 cells. Individual histograms showing surface marker expression of CD40 and MHC-II on DC2.4 cells following 24-hour *in vitro* treatment, analyzed by flow cytometry. Fluorescence intensity is compared to unstained controls.

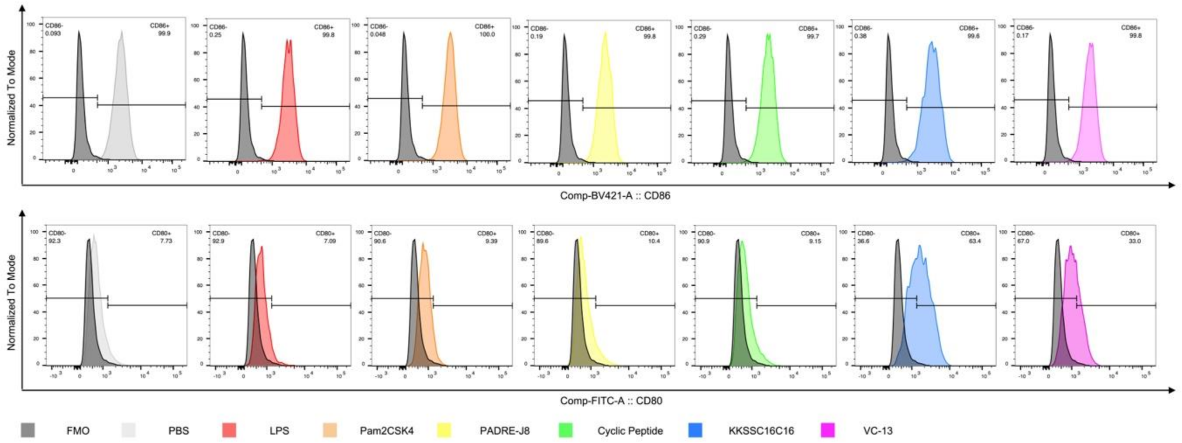

Fig. S17 Representative histograms of CD86 and CD80 expression in DC2.4 cells. Individual histograms showing surface marker expression of CD86 and CD80 on DC2.4 cells after 24 hr *in vitro* treatment, analysed by flow cytometry. Fluorescence minus one (FMO) controls were used to set gating thresholds.

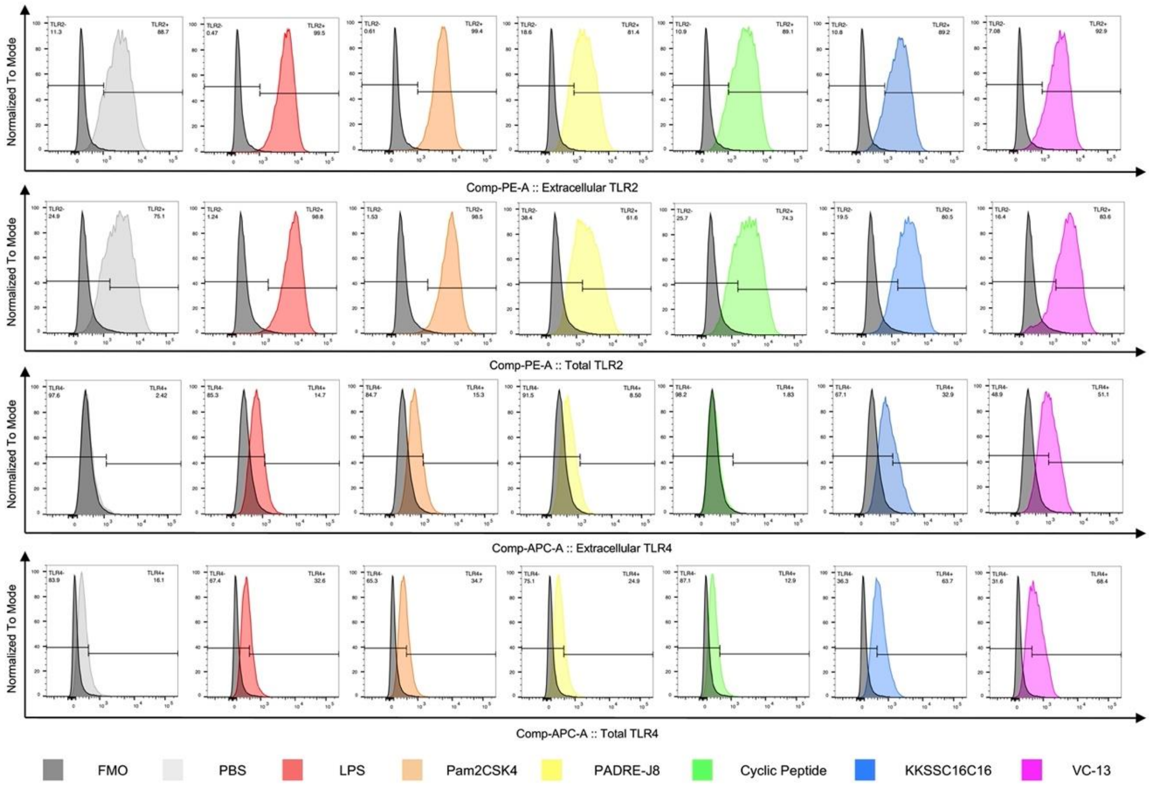

Fig. S18 Representative histograms showing the expression of toll-like receptors 2 and 4 in DC2.4 cells.

Individual histograms represent TLR2 and TLR4-mediated expression following a 24 hr *in vitro* treatment, both extracellularly and intracellularly.

Table S1. Schematic illustration of vaccine formulations.

The table outlines the vaccine names, corresponding adjuvants, and antigens evaluated for immune response in mice. It includes peptide antigens from group A *Streptococcus* (J8, NS1 and 88/30) and GnRH, protein antigens (BSA and SARS-CoV-2 RBD) and the small-molecular hapten physically mixed vaccines assessed for their adjuvant activity.

Vaccine Name	Adjuvant	Antigen
VC-13	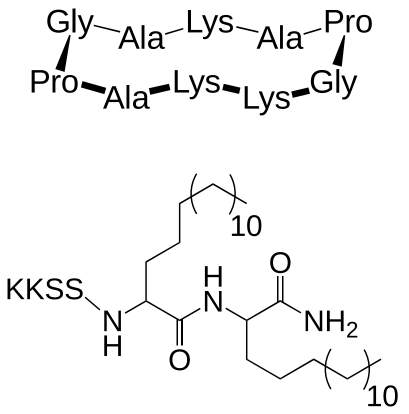	PADRE J8
VC-19		NS1 PADRE
VC-20		PADRE NS1
VC-21		88/30 PADRE
VC-22		PADRE 88/30
VC-23		PADRE GnRH
VC-24		BSA
VC-25		RBD
VC-26		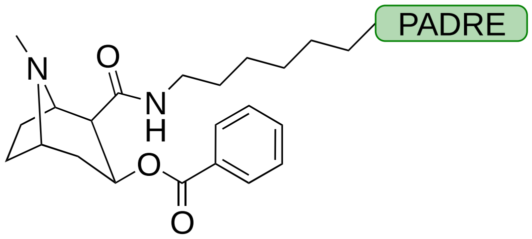

J8 B cell antigen (QAEDKVKQSREAKKQVEKALKQLEDKVQ); NS1 (emm100 antigen; RVTTRSQAQDAAGLKEKAD); 88/30 (emm97 antigen; DNGKAIYERARERALQELGP); GnRH (EHWSYGLRPG); PADRE T helper epitope (AKFVAAWTLKAAA). BSA: bovine serum albumin which is a *serum albumin protein derived from cows. RBD: SARS-CoV-2 RBD which is the receptor binding domain (RBD) of the SARS-CoV-2 protein responsible for COVID-19 infection. HAP: Small molecule hapten from cocaine. Positive controls: antigen (J8, NS1, 88/30, GnRH, BSA, RBD and PADRE-hapten [HAP])-PADRE +/– CFA. Negative control: PBS. 'VC' represents 'vaccine candidate'.*

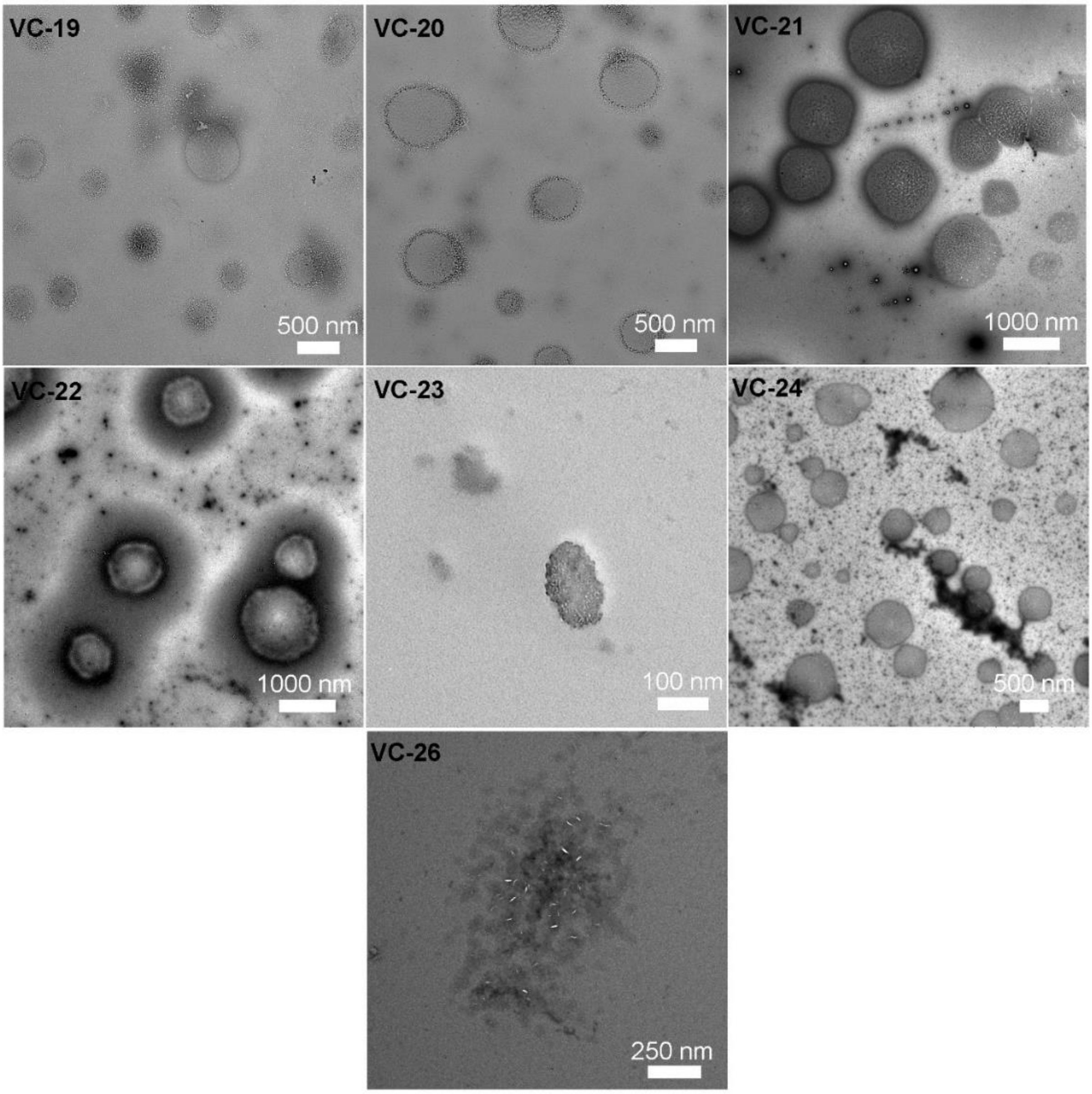

Fig. S19 Transmission electron microscopy (TEM) of VC-19, VC-20, VC-21, VC-22, VC-23, VC-24 and VC-26 (0.1 mg/mL).

Rulers indicate scales of 100 nm, 250 nm, 500 nm or 1000 nm individually. Negative staining was performed using 2% uranyl acetate. Due to research costs associated with the SARS-CoV-2 RBD protein, TEM analysis of the SARS-CoV-2 RBD vaccine (**VC-25**) was not conducted in this study.

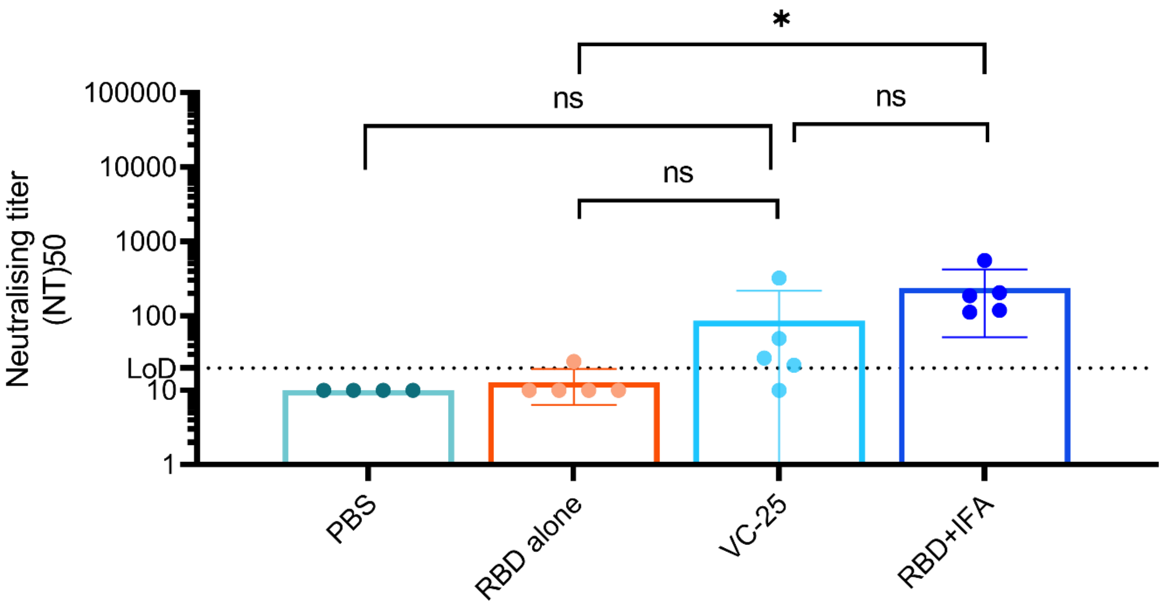

Fig. S20 Pseudovirus neutralization assay.

The (NT)50 indicates the concentration of antibody required to neutralize 50% of the SARS-CoV-2 pseudovirus.
